# Data-Driven Mathematical Model of Osteosarcoma

**DOI:** 10.3390/cancers13102367

**Published:** 2021-05-14

**Authors:** Trang Le, Sumeyye Su, Arkadz Kirshtein, Leili Shahriyari

**Affiliations:** 1 Department of Mathematics and Statistics, University of Massachusetts Amherst, Amherst, MA 01003, USA; tramle@umass.edu (T.L.); sumeyyesu@umass.edu (S.S.); 2 Department of Mathematics, Tufts University, Medford, MA 02155, USA; Arkadz.Kirshtein@tufts.edu

**Keywords:** osteosarcoma, data-driven mathematical model, immune variations, sensitivity analysis, gene expression profiles, tumor deconvolution, immune interactions, tumor microenvironment

## Abstract

**Simple Summary:**

Osteosarcoma is the most common primary bone tumor and has a poor prognosis. Therefore, it is important to understand the mechanism of the development of osteosarcoma to overcome therapy resistance. Several mathematical models have been developed to study the initiation and progression of many cancer types. However, there are currently no mathematical models for the progression of osteosarcoma, to the best of our knowledge. In this work, we develop a data-driven mathematical model to analyze the impact of the immune cell interactions on the growth of osteosarcoma tumors that have distinct immune patterns. Our model provides a foundation for investigating the effect of various treatments on the dynamics of key players in the primary tumor, including immune cells and cytokines, and ultimately the whole tumor.

**Abstract:**

As the immune system has a significant role in tumor progression, in this paper, we develop a data-driven mathematical model to study the interactions between immune cells and the osteosarcoma microenvironment. Osteosarcoma tumors are divided into three clusters based on their relative abundance of immune cells as estimated from their gene expression profiles. We then analyze the tumor progression and effects of the immune system on cancer growth in each cluster. Cluster 3, which had approximately the same number of naive and M2 macrophages, had the slowest tumor growth, and cluster 2, with the highest population of naive macrophages, had the highest cancer population at the steady states. We also found that the fastest growth of cancer occurred when the anti-tumor immune cells and cytokines, including dendritic cells, helper T cells, cytotoxic cells, and IFN-γ, switched from increasing to decreasing, while the dynamics of regulatory T cells switched from decreasing to increasing. Importantly, the most impactful immune parameters on the number of cancer and total cells were the activation and decay rates of the macrophages and regulatory T cells for all clusters. This work presents the first osteosarcoma progression model, which can be later extended to investigate the effectiveness of various osteosarcoma treatments.

## 1. Introduction

Osteosarcoma is the most common form of bone malignancy, which is a rare type of cancer with about 1000 new cases diagnosed each year in the United States [1]. Osteosarcoma has a bimodal age distribution, with the first peak in the 10–14-year-old range and the second peak in adults older than 65 years [2,3]. Past treatments with radiotherapy or anticancer drugs and having heritable syndromes and certain conditions, such as Li-Fraumeni syndrome, hereditary retinoblastoma, and Bloom and Werner syndromes, are considered as risk factors, and surgery, chemotherapy, radiation therapy, and targeted therapy are the types of standard treatment for osteosarcoma [4].

Despite improved outcomes from neoadjuvant chemotherapy in the treatment of osteosarcoma, the average survival of patients with metastasis has remained poor over the last three decades [5,6,7]. Immunotherapy and targeted therapy have recently demonstrated significant results in the treatment of certain cancer types [8,9]. Although these are also popular alternative treatments for osteosarcoma, they are still ineffective for many patients [10]. Osteosarcoma tumors have also been reported to be resistant to the radiotherapy [11,12]. For this reason, a novel technique, hyperthermia, has been developed to increase the effectiveness of radiation [13,14,15,16]. There are some studies that focus on hyperthermia to optimize the treatments for osteosarcoma [17,18]. However, there is of yet no mathematical model that focuses on the tumor microenvironment to provide insights on how to increase the effectiveness of these treatments. Therefore, it is important to investigate the osteosarcoma tumor microenvironment to understand the variability in response to these treatments to overcome therapy resistance [19].

Several studies have shown that cancer cells and tumor infiltrating immune cells (TIICs) play a key role in tumor progression and the identification of malignant tumor types [20,21,22]. Research found that innate immune cells contribute to tumor suppression in several ways, such as recognition and killing of cancer cells [23]. The immune response in the cancer microenvironment can be triggered by tumor antigen detection by immature dendritic cells, which then mature into dendritic cells [24]. Dendritic cells present these antigens to helper and cytotoxic T cells, leading to their activation and the direct killing of cancer by cytotoxic cells [25,26,27]. Helper T cells and cytotoxic T cells also produce IFN-γ that inhibits tumor growth [27,28,29].

On the other hand, certain immune cells have promoting or dual effects on cancer progression. Regulatory T cells inhibit the differentiation and activities of helper and cytotoxic T cells, thus, indirectly promoting tumor by suppressing the immune response [26,29,30,31]. Macrophages, the most abundant immune cells in many cancers, have anti-tumor properties by activating helper and cytotoxic T cells through IL-12 and IL-23 production [26,29,32,33] and also have pro-tumor properties through secreting IL-6, which supports cancer cell proliferation [32,34,35,36,37]

The relationship between clinical outcome and immune cells in osteosarcoma has been found in many studies. Cytotoxic T cells are the primary effector cells of adaptive immunity targeting osteosarcoma [27], and they were found to play a significant role in the immune responses of osteosarcoma patients [38]. Treatments using the antitumor immunocompetence of innate immune cells, such as NK cells and γδ T cells, have been shown to be effective for osteosarcoma tumors [39,40]. Accumulating evidence demonstrates the critical roles of the relative abundance of various immune cells and their interaction network in the initiation and development of osteosarcoma tumors.

There are many studies that use mathematical models to explain the dynamics of tumor growth, to develop clinical responses, to identify the right therapy combination, and to overcome drug resistance in various cancer types [41,42,43,44,45,46,47,48,49]. Although some studies include bone modeling, osteoblast cells, or osteosarcoma treatments [50,51,52,53,54], to the best of our knowledge, there is currently no mathematical model explaining the progression of osteosarcoma tumors. The relationship between immune cells and tumor cells have been used as an alternative approach in the mathematical modeling of different cancers types in some studies [55,56,57,58]. Objective of this study is to build a data-driven model for the progression of osteosarcoma tumors that considers immune cell interactions with tumor cells.

**Figure 1:**
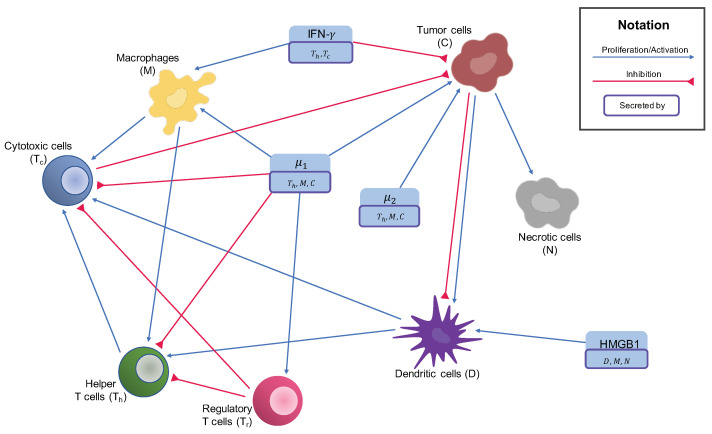
**Interaction network of the tumor microenvironment in osteosarcoma**. Activations and proliferations are shown by blue arrows, and inhibitions are indicated by red arrows.

We recently found that there are three distinct groups of immune patterns of osteosarcoma primary tumors through estimating immune cell proportions by applying a tumor deconvolution method on primary tumor gene expression profiles [59]. In this study, we develop a data-driven mathematical model of osteosarcoma based on the network given in Figure 1 and use a system of ordinary differential equations (ODEs) to represent the interactions.

We then investigate the differences in the tumor growth of patients belonging in three distinct groups of immune patterns, which are obtained by clustering patients based on their immune profiles. We calculate the patient-specific parameters from data in each group to generate “virtual patients” to use in the mathematical model. Lastly, we analyze the dynamics of tumors in each group to find relationships that could be used to explain the effects of the tumor microenvironment on the progression of osteosarcoma tumors.

## 2. Materials and Methods

We built a kinetic model based on the key interactions between the immune system and osteosarcoma cells. In particular, we utilized a system of ordinary differential equations to study the changes in population of the various components of tumor microenvironment throughout time in units of days. To avoid too much complexity, we did not model the spatial distributions of these variables, chemotaxis, or other non-linear phenomena. For biochemical processes A+B→C, we apply the mass action law $\frac{dC}{dt}=\lambda AB$, where λ is the production rate of *C* from *A* and *B*. For all the equations in our model, the symbol λ denotes proliferation, activation, or production rates, and the symbol δ denotes inhibition, decay, or death rates. The variables in the model are given in Table 1 and their interactions are illustrated in Figure 1.

### 2.1. Cytokines

We modeled the dynamics of cytokines through the rate at which they are produced and their natural decay. We assumed that cytokine production rates are proportional to the population of cells that produce them, similar to [60], and that cytokine decay rates are proportional to their own population, which is a common approach [60,61,62,63,64]. In order to simplify the system of equations, we combine some cytokines with similar functions and use the quasi-steady state assumption on other cytokines.

We combine TGF-β, IL-4, IL-10, and IL-13 as $\mu_{1}$. TGF-β and IL-10 are secreted by helper T cells, M2 macrophages, and cancer cells [32,33,35,65,66,67]. IL-4 and IL-13 are secreted by helper T cells and M2 macrophages [32,65,68]. Thus, we model the dynamics of $\mu_{1}$ as:

(1) $$\frac{d[\mu_{1}]}{dt}=\lambda_{\mu_{1}T_{h}}\left[T_{h}\right]+\lambda_{\mu_{1}M}\left[M\right]+\lambda_{\mu_{1}C}\left[C\right]-\delta_{\mu_{1}}\left[\mu_{1}\right]$$

$\mu_{2}$ consists of IL-6 and IL-17, where IL-6 is produced by M1 macrophages, helper T cells, and cancer cells [33,36,65,67,69], and IL-17 is produced by helper T cells [32]. The corresponding equation for $\mu_{2}$ is:

(2) $$\frac{d[\mu_{2}]}{dt}=\lambda_{\mu_{2}T_{h}}\left[T_{h}\right]+\lambda_{\mu_{2}M}\left[M\right]+\lambda_{\mu_{2}C}\left[C\right]-\delta_{\mu_{2}}\left[\mu_{2}\right]$$

IFN-γ is secreted by helper T cells, cytotoxic T cells, and natural killer cells [29,32,70]. As a result, the equation for IFN-γ is written as:

(3) $$\frac{d[I_{\gamma }]}{dt}=\lambda_{I_{\gamma }T_{h}}\left[T_{h}\right]+\lambda_{I_{\gamma }T_{c}}\left[T_{c}\right]-\delta_{I_{\gamma }}\left[I_{\gamma }\right]$$

HMGB1 is passively released by necrotic cells [25,71,72,73] and actively released by macrophages and dendritic cells [71,72,74,75,76,77], leading to the following equation:

(4) $$\frac{d[H]}{dt}=\lambda_{HM}\left[M\right]+\lambda_{HD}\left[D\right]+\lambda_{HN}\left[N\right]-\delta_{H}\left[H\right]$$

We use the quasi-equilibrium state assumption on the other cytokines and estimate them to be proportional to the number of cells that produce them. IL-12 and IL-23 are both secreted by M1 macrophages and dendritic cells [29,32,65,66,67,78]; therefore, we model the concentration of these cytokines as:

(5) $$\begin{array}{cc} \left[\mathrm{IL}\text{-}12\right] & \approx c_{1}\times \left[M\right]+c_{2}\times \left[D\right] \end{array}$$

(6) $$\begin{array}{cc} \left[\mathrm{IL}\text{-}23\right] & \approx c_{3}\times \left[M\right]+c_{4}\times \left[D\right] \end{array}$$

where $c_{1},c_{2},c_{3}$, and $c_{4}$ are constants.

### 2.2. Cells in the Tumor Microenvironment

Since mature immune cells are differentiated from naive immune cells, we model the population of each mature immune cell to be proportional to its respective naive immune cell, where the proportion is determined by the cells/cytokines that activate the naive cells. Similar to the cytokine equations, for each mature immune cell, we also include a natural death rate $\delta_{\mathrm{cell}}$.

#### 2.2.1. Macrophages

Since macrophages have many phenotypes and are constantly changing their phenotype, we model all macrophages together as one variable to avoid overly great complexity. M1 and M2 macrophages are differentiated from naive macrophages or monocytes. M1 macrophages are activated by IFN-γ [35,66,67], while M2 macrophages are activated by IL-4, IL-10, and IL-13 [33,66,67,79], where IL-4, IL-10, and IL-13 belong to $\mu_{1}$. Therefore, we can write the dynamics of macrophages as:

(7) $$\frac{d[M]}{dt}=\left(\lambda_{MI_{\gamma }}\left[I_{\gamma }\right]+\lambda_{M\mu_{1}}\left[\mu_{1}\right]\right)\left[M_{N}\right]-\delta_{M}\left[M\right]$$

By taking into account the activations from Equation (7) and introducing the independent naive macrophage/monocyte production parameter $A_{M_{N}}$, we have the equation for naive macrophages/monocytes:

(8) $$\frac{d[M_{N}]}{dt}=A_{M_{N}}-\left(\lambda_{MI_{\gamma }}\left[I_{\gamma }\right]+\lambda_{M\mu_{1}}\left[\mu_{1}\right]\right)\left[M_{N}\right]-\delta_{M_{N}}\left[M_{N}\right]$$

#### 2.2.2. T Cells and NK Cells

We model the following subtypes of T cells: helper T cells, regulatory T cells, and cytotoxic cells, where cytotoxic cells include cytotoxic T cells and natural killer cells.

Helper T cells are activated by dendritic cells, IL-12, and IL-23 [26,29,32,80], and are inhibited by regulatory T cells, IL-10, and TGF-β [29,31,81,82], resulting in the equation:

(9) $$\frac{d[T_{h}]}{dt}=\left(\lambda_{T_{h}M}\left[M\right]+\lambda_{T_{h}D}\left[D\right]\right)\left[T_{N}\right]-\left(\delta_{T_{h}T_{r}}\left[T_{r}\right]+\delta_{T_{h}\mu_{1}}\left[\mu_{1}\right]+\delta_{T_{h}}\right)\left[T_{h}\right]$$

Regulatory T cells are activated by IL-10 and TGF-β [29,83], hence their dynamics are modeled by:

(10) $$\frac{d[T_{r}]}{dt}=\lambda_{T_{r}\mu_{1}}\left[\mu_{1}\right]\left[T_{N}\right]-\delta_{T_{r}}\left[T_{r}\right]$$

Cytotoxic cells (cytotoxic T cells and NK cells) are activated by helper T cells, dendritic cells and IL-12 [25,26,27,29,33,84,85] and are inhibited by regulatory T cells, IL-10, and TGF-β [26,30,67,83]. The corresponding equation is:

(11) $$\frac{d[T_{c}]}{dt}=\left(\lambda_{T_{c}T_{h}}\left[T_{h}\right]+\lambda_{T_{c}M}\left[M\right]+\lambda_{T_{c}D}\left[D\right]\right)\left[T_{N}\right]-\left(\delta_{T_{c}T_{r}}\left[T_{r}\right]+\delta_{T_{c}\mu_{1}}\left[\mu_{1}\right]+\delta_{T_{c}}\right)\left[T_{c}\right]$$

Combining all the activations from Equations (9)–(11) as well as adding parameter $A_{T_{N}}$ for the independent production rate of naive T cells, we obtain the equation for naive T cells:

(12) $$\begin{array}{cc} \frac{d[T_{N}]}{dt}= & A_{T_{N}}-\left(\lambda_{T_{h}M}\left[M\right]+\lambda_{T_{h}D}\left[D\right]\right)\left[T_{N}\right]-\lambda_{T_{r}\mu_{1}}\left[\mu_{1}\right]\left[T_{N}\right]\\ \; & -\left(\lambda_{T_{c}T_{h}}\left[T_{h}\right]+\lambda_{T_{c}M}\left[M\right]+\lambda_{T_{c}D}\left[D\right]\right)\left[T_{N}\right]-\delta_{T_{N}}\left[T_{N}\right] \end{array}$$

#### 2.2.3. Dendritic Cells

Dendritic cells are activated by cancer cells and HMGB1 [26,71,74,76,77]. However, cancer cells can also promote apoptosis in dendritic cells through many tumor-derived factors, such as gangliosides, neuropeptides, etc. [78]. By introducing the independent production rate of naive dendritic cells $A_{D_{N}}$, we can describe the dynamics of naive and mature dendritic cells with the following system:

(13) $$\frac{d[D]}{dt}=\left(\lambda_{DC}\left[C\right]+\lambda_{DH}\left[H\right]\right)\left[D_{N}\right]-\left(\delta_{DC}\left[C\right]+\delta_{D}\right)\left[D\right]$$

(14) $$\frac{d[D_{N}]}{dt}=A_{D_{N}}-\left(\lambda_{DC}\left[C\right]+\lambda_{DH}\left[H\right]\right)\left[D_{N}\right]-\delta_{D_{N}}\left[D_{N}\right]$$

#### 2.2.4. Cancer Cells

Osteosarcoma cells are typically of osteoblastic origin and are characterized by abnormally high proliferation and low apoptosis. We denote the high proliferation rate of cancer cells as $\lambda_{C}$.

Osteosarcoma growth is promoted by IL-6, IL-17, and TGF-β [29,34,35,36,37,69,86,87]. Tumor cells are killed by cytotoxic cells [26,88,89], while their growth is inhibited by IFN-γ [26,27,70]. In the mathematical modeling of cancer, it is common to estimate the growth to be proportional to $\left[C\right]\left(1-\frac{\left[C\right]}{C_{0}}\right)$, where $C_{0}$ is the carrying capacity [90,91]. As a result, we have the following equation for cancer cells:

(15) $$\begin{array}{cc} \frac{d[C]}{dt}= & \left(\lambda_{C}+\lambda_{C\mu_{1}}\left[\mu_{1}\right]+\lambda_{C\mu_{2}}\left[\mu_{2}\right]\right)\left[C\right]\left(1-\frac{\left[C\right]}{C_{0}}\right)\\ \; & -\left(\delta_{CT_{c}}\left[T_{c}\right]+\delta_{CI_{\gamma }}\left[I_{\gamma }\right]+\delta_{C}\right)\left[C\right] \end{array}$$

#### 2.2.5. Necrotic Cells

Necrotic cells, which are cells that go through the process of necrotic cell death, are promoted by cancer cells since, when cancer cells are killed by cytotoxic cells, a proportion of them become necrotic cells. In particular, the “production” rate of necrotic cells can be modeled as a fraction of the dying cancer cells, resulting in the following dynamics:

(16) $$\frac{d[N]}{dt}=\alpha_{NC}\left(\delta_{CT_{c}}\left[T_{c}\right]+\delta_{CI_{\gamma }}\left[I_{\gamma }\right]+\delta_{C}\right)\left[C\right]-\delta_{N}\left[N\right]$$

### 2.3. Data of the Model

There are some popular tumor deconvolution methods to estimate the relative frequency of cells from the gene expression data of a bulk of cells, and CIBERSORTx B-mode [92] has been shown in recent studies to have the best performance among these methods [93,94]. In our previous study [59], we used the gene expression data sets from two cohorts, TARGET and GSE21257 [95] downloaded from the UCSC Xena web portal [96] and GEO website respectively, to use in CIBERSORTx B-mode to estimate the immune cell frequencies. Then, K-means clustering [97] was applied on the estimated immune cell fractions. The number of clusters for the K-means algorithm was chosen using the elbow method [98]. As a result, we found that there were three distinct immune patterns of osteosarcoma tumors.

**Figure 2:**
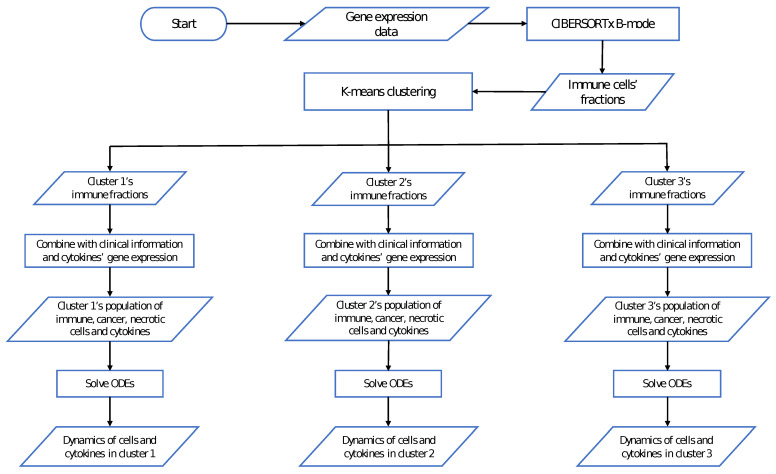
**The general workflow of this study.** Given the gene expression data of tumors, immune cell fractions were estimated using CIBERSORTx B-mode. Then, K-means clustering was applied to find three clusters with distinct immune compositions. For each cluster, the populations of immune, cancer, and necrotic cells were derived from immune fractions and clinical information. These cell populations and cytokine expression levels were used as input (either as the initial conditions or steady states) in the system of ODEs to find the dynamics of the components of the tumor microenvironment in each cluster.

**Figure 3:**
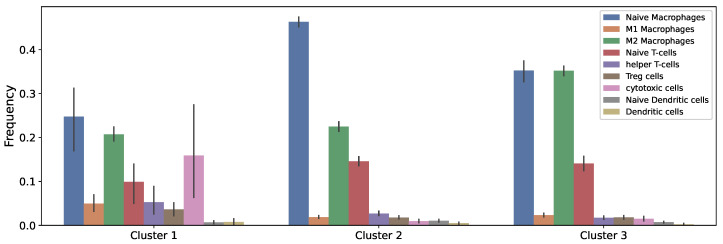
**The immune cell fractions used in the model.** Clusters were derived based on differences in 22 immune cell types of osteosarcoma tumors.

In this study, we used the same cluster assignment for the TARGET data with 88 samples and used our mathematical model to study the dynamics of the tumor microenvironment of each cluster from the initial time of diagnosis until reaching their steady state. The general workflow of this study is described in Figure 2, and the average immune fractions of various cell types in each cluster are shown in Figure 3, where the vertical bars denote the 95% confidence intervals.

The outputs of CIBERSORTx only provide the fractions of each immune cell within the tumor tissue; however, we need the number of immune cells along with the number of cancer and necrotic cells as inputs to our model. Thus, we download the supplementary data of the TARGET project, which has information on the percentage of normal, stroma, tumor, and necrotic cells of each sample. We used the percentage of normal cells to represent the percentage of total immune cells in the sample.

First, we converted the immune cell fractions to the immune cell population by multiplying the fractions with a scaling factor $\alpha_{dim}$. Then, knowing the percentage of total immune cells, cancer cells, and necrotic cells, we derived the population of cancer and necrotic cells from the population of total immune cells. For example, given the total immune population *I*, the cancer and necrotic cell abundance can be calculated as

(17) $$\begin{array}{c} C=I\times \frac{\%\;\mathrm{of}\;\mathrm{cancer}\;\mathrm{cells}}{\%\;\mathrm{of}\;\mathrm{total}\;\mathrm{immune}\;\mathrm{cells}} \end{array}$$

(18) $$\begin{array}{c} N=I\times \frac{\%\;\mathrm{of}\;\mathrm{necrotic}\;\mathrm{cells}}{\%\;\mathrm{of}\;\mathrm{total}\;\mathrm{immune}\;\mathrm{cells}} \end{array}$$

where *C* and *N* are the cancer and necrotic cell population, respectively.

To choose a reasonable value for $\alpha_{dim}$, we first estimated the average osteosarcoma tumor volume. We found the mean volume of Ewing sarcomas to be 275 mL based on the tumor volumes given in [99], and Ewing sarcoma has been reported to have a similar volume to osteosarcoma [100]. Thus, we estimated the average osteosarcoma’s volume to be 275 mL.

Osteoblasts, which are the cells of origin of osteosarcoma, have a diameter of 20–50 μm [101]; therefore, we approximated osteosarcoma cells to have an average diameter of 35 μm, resulting in an average of $6.4\times 10^{9}$ osteosarcoma cells in osteosarcoma tumors. We then choose $\alpha_{dim}=1.765\times 10^{8}$ to match the average number of cancer cells among all patients in our data to $6.4\times 10^{9}$ cells. However, it is important to note that $\alpha_{dim}$ is simply a scaling factor and does not have any effects on the dynamics of cells or on the relative cell abundance between clusters.

### 2.4. Parameter Estimation

Some parameters of our model, such as the decay/death rates of immune cells and cytokines, were taken from available research (more details in Appendix B.1), while others were estimated. We follow the common approach from mathematical biological models to use assumptions on the steady state values of the system to derive those unknown parameters [102,103]. In particular, we make the assumption that after a tumor reaches a very large size, the immune variation within the tumor microenvironment is minuscule, and we denote this state as the steady state of our system.

Different immune patterns of tumors, such as high or low levels of helper and cytotoxic T cells in one group versus another group, indicate that the activation rates of different T cell sub-types from naive T cells vary from one group of tumors to another group. Hence, many parameters of the model, such as the activation rates of T cell sub-types, depend on the tumor immune profile, and therefore we estimated the parameters separately for each cluster.

We assumed the samples with a large number of cancer cells were at the steady state. For each cluster, we used the 85th percentile of cancer abundance as the cutoff, and calculated the steady state values for the cluster by averaging the values from samples that had more cancer cells than this cutoff. Table 2 shows the steady state values of every cluster.

Our assumption above asserts that the rate of change of our model’s variables is 0 at the steady state, or equivalently $\frac{dX}{dt}=0$ at the steady state. With the additional assumptions in Appendix B.1, as well as knowing the steady state values of our model’s variables, we can derive parameter values for each cluster using the fsolve function from the SciPy package in Python. The parameter values for each cluster are given in Table A1.

### 2.5. Non-Dimensionalization

To remove the scale dependence and obtain additional numerical stability, we applied non-dimensionalization on all equations of our system. For a model variable *X* converging to the steady state value $X^{\infty }$, we created a non-dimensional variable $\bar{X}$ such that $\bar{X}=\frac{X}{X^{\infty }}$. Then, $\bar{X}$ satisfies the equation $\frac{d\bar{X}}{dt}=F\left(\bar{X},\bar{\theta },t\right),$ where $\bar{\theta }$ is the vector of non-dimensional parameters. The full system of non-dimensionalized equations are given in Appendix C.

To solve the non-dimensional dynamical system for each cluster, we applied the odeint function from the SciPy package [104], with the initial conditions from a data point of interest from the TARGET data set.

### 2.6. Sensitivity Analysis

To evaluate the quality of our parameters through how they affect the dynamics of the system, we performed a global gradient-based sensitivity analysis on all parameters of our system.

For the non-dimensional system $\frac{d\bar{X}}{dt}=F\left(\bar{X},\theta ,t\right)$ with *N* parameters $\theta =\theta_{1},\ldots ,\theta_{N}$, the (first order) sensitivity $s_{i}$ of parameter $\theta_{i}$ was defined as the gradient of the model output with respect to the parameter [105]:

(19) $$\begin{array}{c} s_{i}=\frac{d\bar{X}}{d\theta_{i}} \end{array}$$

We calculated the sensitivity $s_{i}$ for each parameter at the steady state of the equation for two quantities of interest: cancer cell abundance and total cell abundance. Consider the general steady state system as $F(X^{*},\theta )=0,$ with $X^{*}$ being the equilibrium values of our model’s variables. The sensitivity vector *s* can be obtained analytically by differentiating the steady-state equation with respect to parameter vector θ, that is,

(20) $$\begin{array}{c} \nabla F\left(X^{*},\theta \right)\frac{dX^{*}}{d\theta }+\frac{\partial F(X^{*},\theta )}{\partial \theta }=0 \end{array}$$

where $\nabla F(X^{*},\theta )$ is the Jacobian matrix of $F(X^{*},\theta )$ with respect to *X*. Then, to compute sensitivity vector *s* at equilibrium, or equivalently $\frac{dX^{*}}{d\theta }$, we simply need to numerically invert $\nabla F(X^{*},\theta )$.

Generally, $s_{i}$ varies for different values of the parameter set; thus, we define the local sensitivity $S_{i}$ of parameter $\theta_{i}$ for a chosen neighborhood Ω(θ) of the given parameter set as

(21) $$\begin{array}{c} S_{i}=\int_{\Omega }s_{i}\left(\theta \right)d\theta \end{array}$$

where the integral is evaluated numerically using sparse grid points [106,107].

Since we made many assumptions to derive the parameter values for our model and different assumptions can lead to different parameter values, we vary these assumptions by a scaling factor of 0.01 to 100 for *K* times and obtain the local sensitivity $S_{i}^{k}$, with k=1,…,K, for parameter $\theta_{i}$ derived from the $k^{\mathrm{th}}$ set of new assumptions. Then, the global sensitivity $S_{i}$ of parameter $\theta_{i}$ is a weighted average of the local sensitivities $S_{i}^{k}$ for k=1,…,K:

(22) $$\begin{array}{c} S_{i}=\sum_{k=1}^{K}w_{k}S_{i}^{k} \end{array}$$

where $w_{k}$ is chosen so that the parameter values that are closer to the original parameter set have larger weights and the parameter values that are very different from the original parameter set have smaller weights. This method of choosing $w_{k}$ is based on the idea of the weighted average of local sensitivities in [105].

## 3. Results

We obtained the dynamics of the components in the tumor microenvironment by solving the above mentioned system of ODEs with parameters derived from the cancer patient data using the steady state assumption as mentioned in Section 2.4. Given non-negative initial conditions and non-negative parameters, the solution of the systems remains non-negative and globally bounded (Appendix A.2 and Appendix A.3).

### 3.1. Dynamics of the Tumor Microenvironment

We are interested in exploring the dynamics of different components of the osteosarcoma microenvironment as well as the difference in cancer progression between clusters. Hence, we want to model the dynamics with similar initial cancer populations among clusters. We first choose the sample with the smallest cancer population in cluster 1, and then choose a sample from cluster 2 and 3 that has the most similar cancer population to the chosen sample in cluster 1. We use these samples as the initial conditions for their corresponding cluster. Table 3 shows the dimensionless initial condition values of each cluster.

We observe that, as the cancer population grows, helper T cells, dendritic cells, cytotoxic cells, and IFN-γ populations first increase and then decrease over time. This makes sense since, in the early stage of cancer, naive dendritic cells come in contact with tumor antigens, inducing the activation and increase in the number of dendritic cells [24,26]. Dendritic cells present tumor antigens to helper T cells and cytotoxic cells and activate them [108], resulting in an increase of these cells. Helper T cells and cytotoxic cells then produce IFN-γ [29,32,70], leading to this cytokine’s increased abundance. As the tumor grows bigger, some cancer cells develop variants that are resistant to detection by naive dendritic cells [109], and thus the number of dendritic cells finally decreases, eventuating in the decrease in helper T cells and cytotoxic cells, and accordingly the decrease in IFN-γ. This is likely the escape phase of immunoediting, when cancer cells escape the immune system and outgrow the immune cells.

The switch in dynamics from increasing to decreasing in dendritic cells, helper T cells, cytotoxic cells, and IFN-γ occurs around the same time that cancer cells start growing fast. Contrastingly, the number of regulatory T cells decreases when these cells increase and increases when these cells decrease. Hence, regulatory T cells start increasing in density when the tumor is at its peak of growing. Regulatory T cells have the role of modulating the immune system and consequently promote tumor growth; therefore, we can expect the opposite dynamics to anti-tumor immune cells and cytokines, such as dendritic cells, helper T cells, cytotoxic cells, and IFN-γ. In general, it is important to study this switch in the dynamics since it can be used as the predictor of the highest growth of cancer cells during tumor development.

On the other hand, the macrophage population first decreases and then increases during osteosarcoma progression, while necrotic cells, HMGB1, along with the cytokine groups $\mu_{1}$ and $\mu_{2}$ increase in population as cancer cells grow. As both $\mu_{1}$ and $\mu_{2}$ support tumor growth, their population growth over time could contribute to the fast progression of osteosarcoma. Necrotic cells are mainly cancer cells that were killed by cytotoxic cells or IFN-γ; thus, it is reasonable to see their population grow over time. As a result, HMGB1, which is largely produced by necrotic cells, increases in abundance as the tumor progresses.

Cluster 2’s cancer cells begin by growing more slowly than cluster 1; however, at around 500 days, they start growing very fast and end up having the highest cancer population at the steady state out of all clusters. Our previous study [59] based on the clinical information of the TARGET dataset also indicates that patients in cluster 2 had the worst survival outcomes among the three clusters.

**Figure 4:**
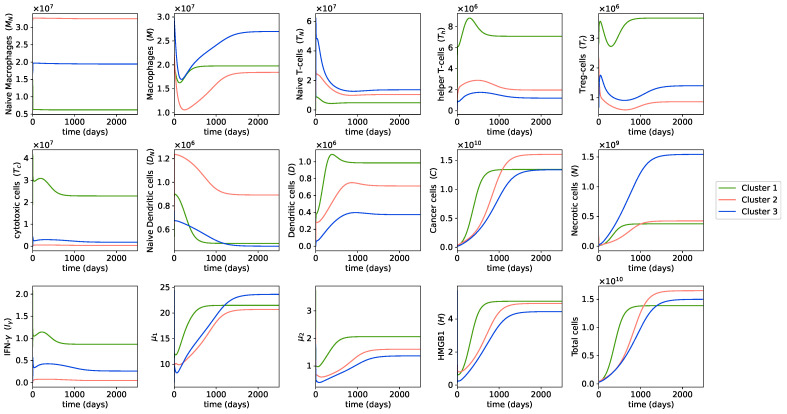
**Dynamics of cells and cytokines in osteosarcoma tumors**. Evolution of the cells and cytokine population in the model is plotted over the time in units of days. This figure shows the dynamics of the variables of the model starting from the time of the first diagnosis of small tumors in each cluster until reaching their steady state values, i.e., the average values of the largest tumors in the same cluster. The different color lines describe the dynamics of different clusters.

Figure 4 shows that cluster 2 had the lowest number of cytotoxic cells, macrophages, and IFN-γ and the highest number of naive macrophages during tumor progression. A high population of cytotoxic cells and IFN-γ are generally associated with a good prognosis because they directly kill cancer cells, while a high level of naive macrophages have been found in our previous study to associate with poor prognosis [59]. Cluster 2 also had the slowest growth rate of necrotic cells. A high number of necrotic cells means many cancer cells have been killed by the immune system and is an indication of a good prognosis. Thus, cluster 2, with a slow growth rate of necrotic cells, high growth rate of cancer, and the highest cancer population at the steady state, had a poor prognosis based on our model’s dynamics.

Cluster 3 had the slowest cancer growth rate among all clusters and a smaller cancer population at the steady state compared with cluster 2. Cluster 3’s necrotic cells had the fastest growth rate and the highest population at the steady state out of the 3 clusters. Hence, the dynamics of cluster 3 appear to be the most favorable. This is in agreement with the findings on the survival outcomes of cluster 3 in our previous study [59].

Cluster 3 had the smallest amount and the slowest growth rate of the cytokine group $\mu_{2}$, which has tumor-promoting effects, both initially and at the steady state (Figure 4). Interestingly, cluster 3 also had the lowest population of helper T cells and dendritic cells over time. These two cells are known to correlate with good prognoses. If we were to simply look at the immune composition of the patients in cluster 3, we might make the wrong prediction on their prognosis due to the low abundance of certain immune cells with good prognostic values. Therefore, it is important to take into consideration the interaction between immune cells and cancer cells, and investigate the dynamics of cancer in addition to studying the immune composition.

Cluster 1 had a high cancer growth rate from the beginning and thus its cancer population reached the steady state faster than the other clusters. However, its cancer cells did not reach as high population at the steady state as the cancer cells in cluster 2. Cluster 1 had the highest levels of both immune cells and cytokines with good prognoses, including cytotoxic cells, helper T cells, dendritic cells, and IFN-γ, and those with poor prognoses, such as regulatory T cells and $\mu_{2}$ during tumor progression. Thus, it is again necessary to look at the interactions within the tumor microenvironment for such clusters.

We observed that $\mu_{1}$ and $\mu_{2}$ grew fast and reached the steady states very quickly in cluster 1. Since both $\mu_{1}$ and $\mu_{2}$ promote tumor proliferation, this could be the reason why cancer cells quickly reach the steady state in cluster 1. Overall, since cluster 1 has a lower cancer population at the steady state compared with cluster 2 but a higher cancer growth rate than cluster 3, its cancer dynamics are worse than cluster 3 but better than cluster 2, which aligns with the results of our previous study [59].

### 3.2. Sensitivity Analysis

We performed global sensitivity analysis with parameters derived from patient data with the steady state assumption in each cluster. The sensitivity analysis was performed on the dimensionless system, and evaluated at the steady states. We were interested in finding which parameters in our system strongly affected the growth of tumors, and thus we used the cancer population and total cell population as variables of interest in the sensitivity analysis.

**Figure 5:**
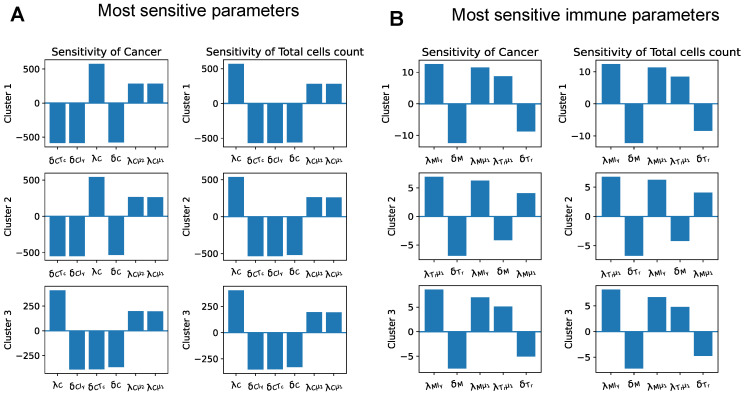
**Sensitivity analysis.** (**A**) The sensitivity level of the most sensitive parameters for cancer and total cell population at the steady state. (**B**) The most sensitive parameters associated with immune cells. The most sensitive parameters for each cluster are shown in each row of plots.

Figure 5A presents the six most sensitive parameters in every cluster. Since we also want to study the effects of the immune system on cancer progression, we looked at the five most sensitive parameters from the immune cells equations as well. Therefore, we plotted the top five most sensitive parameters excluding the parameters from the cancer cell Equation (15) and necrotic cell Equation (16) (Figure 5B).

The most sensitive parameters across the three clusters were the cancer proliferation and inhibition parameters in the cancer Equation (15). As expected, an increase in any of the cancer proliferation parameters ($\lambda_{C},\lambda_{C\mu_{1}},\lambda_{C\mu_{2}}$) resulted in an increase in the number of cancer cells, and an increase in any cancer inhibition parameters ($\delta_{CT_{c}},\delta_{CI_{\gamma }},\delta_{C}$) resulted in a decrease in the number of cancer cells. It is worth noting that all sensitive parameters presented in Figure 5 had similar effects on cancer populations as on total cell populations.

The most sensitive immune parameters were activation and the decay rates of macrophages and regulatory T cells for all clusters. An increase in any activation rates of macrophages and regulatory T cells led to higher cancer and total cell numbers, while an increase in their decay rates caused a decrease in these quantities of interest. This implies that both macrophages and regulatory T cells had tumor-promoting effects.

Since regulatory T cells inhibit helper T cells and cytotoxic cells, they hinder IFN-γ production and, thus, down-regulate cytotoxic cells and IFN-γ’s ability to kill cancer cells. Macrophages, on the other hand, have both anti-tumor phenotype (M1 macrophages) and pro-tumor phenotype (M2 macrophages). However, the predominant portion of macrophages in the patient data across all three clusters was M2 macrophages (Figure 3), which can cause the main effect of macrophages in our model to be pro-tumor.

### 3.3. Dynamics with Varying Assumptions

**Figure 6:**
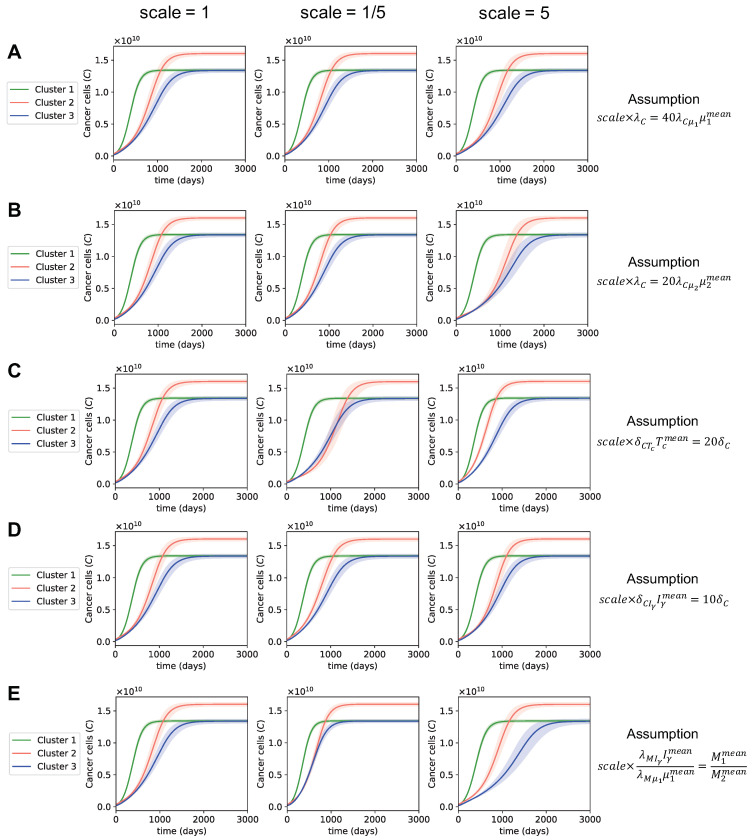
**The dynamics of cancer when the assumptions of the sensitive parameters are varied.** (**A**–**E**) The cancer growth of all three clusters for each assumption of sensitive parameters. The left plot in every sub-figure is the original cancer dynamics, the middle and right plots are the cancer dynamics obtained when the given assumption is scaled by 1/5 and by 5, respectively.

Since we made some assumptions in order to derive the parameter values for each cluster, we wanted to see how the dynamics of cancer population would change when we varied these assumptions. Based on the results of the global sensitivity analysis, we determined that the parameters in the equations of cancer cells, macrophages, and regulatory T cells were the most sensitive parameters. We varied each assumption relating to these sensitive parameters (Equations (A15)–(A19)) by five times in both directions (scale five-times bigger or five-times smaller) and observed how the progression of cancer changed with the new assumptions (Figure 6). For example, since $\lambda_{C}$ and $\lambda_{C\mu_{1}}$ are sensitive parameters, we varied the assumption $\lambda_{C}=40\lambda_{C\mu_{1}}\mu_{1}^{\mathrm{mean}}$ (Equation (A16)) by five times, resulting in the following new assumptions:

(23) $$\begin{array}{c} \lambda_{C}=200\lambda_{C\mu_{1}}\mu_{1}^{\mathrm{mean}},\;\;\;\lambda_{C}=8\lambda_{C\mu_{1}}\mu_{1}^{\mathrm{mean}}, \end{array}$$

where the cancer dynamics with the original assumption (Equation (A16)) is the left plot in Figure 6A (scale = 1), and the cancer dynamics with the new assumptions (Equation (23)) are the middle and right plots in Figure 6A (scale = 1/5 and scale = 5).

We noticed that when we varied the assumptions of the most sensitive parameters, the time for the cancer population to reach the steady state changed by a relatively small amount; however, the overall observation of the cancer dynamics between clusters did not change (Figure 6). That is, these different assumptions led to the same observations: cluster 1’s cancer population reached a steady state the fastest among all clusters, cluster 2’s tumors grew slower than cluster 1’s at first but then began growing fast and resulted in the highest steady state population, and cluster 3 had the most favorable cancer progression with the slowest growth of cancer cells and one of the lowest steady state cancer populations.

The largest changes in the dynamics of cancer were due to the assumptions for the activation rates of macrophages (Figure 6E):

$$\begin{array}{c} \frac{\lambda_{MI_{\gamma }}I_{\gamma }^{\mathrm{mean}}}{\lambda_{M\mu_{1}}\mu_{1}^{\mathrm{mean}}}=\frac{M_{1}^{\mathrm{mean}}}{M_{2}^{\mathrm{mean}}}. \end{array}$$

This assumption was based on the fact that M1 and M2 macrophages are activated by IFN-γ and $\mu_{1}$, respectively, and thus the ratio of macrophages activated by IFN-γ to macrophages activated by $\mu_{1}$ should be approximately equal to the ratio of M1 to M2 macrophages. This is a reasonable assumption that uses patient data to derive the activation rates of macrophages. We expect to see the ground truth ratio of macrophages activated by IFN-γ to macrophages activated by $\mu_{1}$ to be close to our assumption, rather than to differ by five times. Therefore, it is very unlikely to observe cancer dynamics, such as in the middle and right plots in Figure 6E with our data. On the other hand, the assumptions for the death rate of cancer by IFN-γ and the apoptosis rate of cancer, $\delta_{CI_{\gamma }}I_{\gamma }^{\mathrm{mean}}=10\delta_{C}$, appeared to have a negligible to no impact on cancer progression (Figure 6D).

The shaded regions in Figure 6 denote the changes in dynamics when we varied the most sensitive parameters ($\lambda_{C},\lambda_{C\mu_{1}},\lambda_{C\mu_{2}},\delta_{CT_{c}},\delta_{CI_{\gamma }},\delta_{C},\lambda_{MI_{\gamma }},\lambda_{M\mu_{1}},\delta_{M},\lambda_{T_{r}\mu_{1}},$ and $\delta_{T_{r}}$) by 10% in negative and positive directions. We observed that varying the most sensitive parameters by 10% did not create large changes to the cancer dynamics. Overall, Figure 6 shows that, when we change the assumptions of the most sensitive parameters or vary the sensitive parameters themselves, the observations we made about cancer development between clusters in Section 3.1 were not affected. Furthermore, even though several assumptions were made to estimate the parameters, the dynamics of cancer did not greatly depend on these assumptions.

### 3.4. Dynamics with Different Initial Conditions

**Figure 7:**
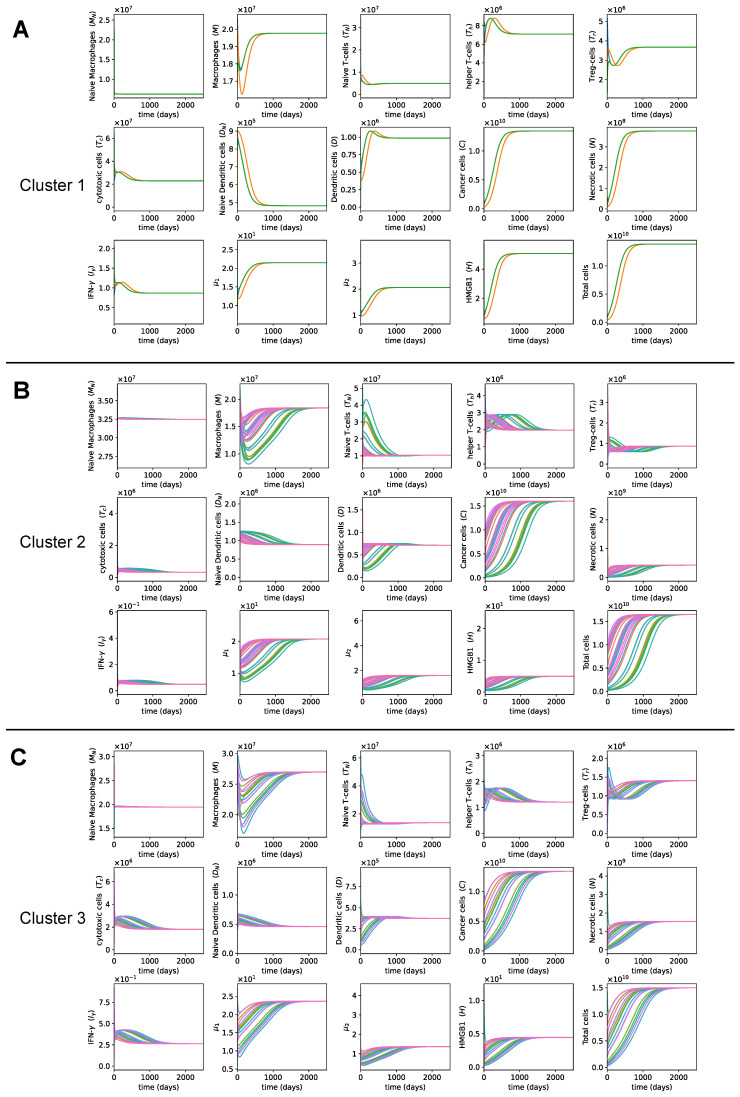
**The dynamics with varying initial conditions.** (**A**–**C**) The dynamics of cells and cytokines with initial conditions from different patients in clusters 1, 2, and 3, respectively.

For each cluster, we also looked at the dynamics with different initial conditions from the different samples within that cluster (Figure 7). We observed that different initial conditions in a cluster led to similar growth patterns of cancer. This makes sense since the dynamics are determined by the parameters of the ODE system, which were derived from the patient data through the steady state assumption in each cluster. As a result, the cancer growth rates and patterns were similar among patients within the same cluster but different among patients in different clusters. Thus, if we know which cluster a patient belongs to, we can predict their cancer growth more accurately than by using the same cancer progression model for all patients.

**Figure A1:**
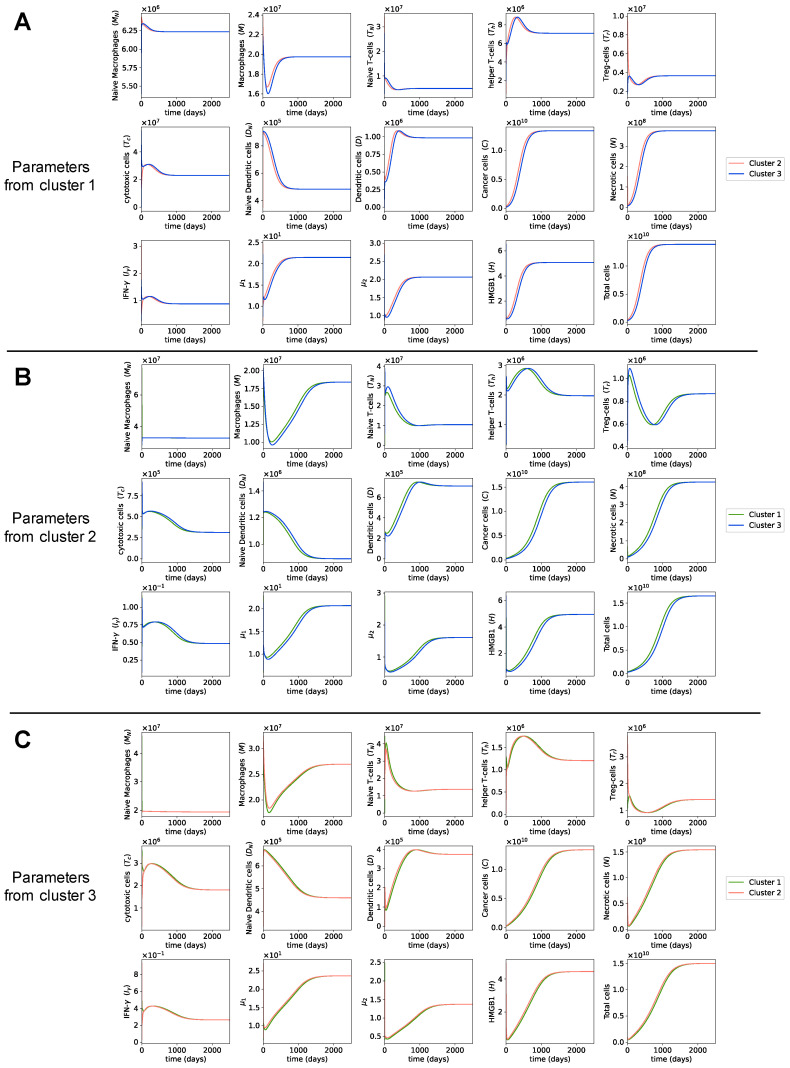
**Dynamics with cross-cluster initial conditions.** (**A**) The dynamics of cells and cytokines with parameters from cluster 1 and initial conditions from clusters 2 and 3. (**B**) The dynamics of cells and cytokines with parameters from cluster 2 and initial conditions from clusters 1 and 3. (**C**) The dynamics of cells and cytokines with parameters from cluster 3 and initial conditions from clusters 1 and 2.

To verify that the parameters in each cluster are what drives the dynamics of the cluster, we examined the dynamics of each cluster with the initial conditions from other clusters (Figure A1 in Appendix D). In particular, we plotted dynamics of cluster 1 with the initial conditions in Table 3 from clusters 2 and 3. These cross-cluster initial conditions quickly converged to the same dynamics, confirming that the dynamics in each cluster were more influenced by the parameters rather than by the initial conditions.

## 4. Discussion

Cancer is a heterogeneous disease with numerous components, such as immune cells, cancer cells, and lymphatic vessels [110], and in typical in vitro and in vivo researches, cancer mechanisms or components are usually studied one at a time. While these experimental studies provide relevant insights about the mechanisms, none of them can provide the adequate required information to understand the complexity of cancer [111]. There are also many mathematical biological papers that model the interactions of immune cells and cancer cells; however, most of them only examine one or two immune cells in their framework [112,113,114,115,116,117,118,119].

A study by Wilkie et al. modeled the combination of all immune cells as one variable and analyzed its effects on tumor growth [120]. However, the impacts of the immune system on cancer are diverse with some immune cells having anti-tumor effects while others had pro-tumor effects, and thus modeling the whole immune system as one variable would fail to capture these important interactions. Only a few papers explore multiple immune cells [61,62,63], but even these models did not investigate the influence of macrophages, which have been shown to be the most abundant cell type in the tumor microenvironment. Moreover, no studies have investigated the interactions between the immune system and cancer cells in osteosarcoma to the best of our knowledge.

The growing availability of biological experimental data and recent advancements in tumor deconvolution methods have increased the demand for data-driven mathematical models that enable us to model different pathways simultaneously and research the system’s complexity more effectively [121]. In this study, we developed the first data-driven mathematical model that takes each tumor’s characteristic into consideration for the progression of an osteosarcoma tumor by utilizing the estimated immune patterns using the gene expression profiles from primary osteosarcoma tumors.

Our results show that, as cancer cells grow in number, the helper T cell, dendritic cell, cytotoxic cell, and IFN-γ populations increase at first and then decrease with time, while regulatory T cells first decrease in population and then increase. This switch in the dynamics of immune cells happens around the time that cancer cells have the fastest growth. The decrease in population of the anti-tumor immune cells and cytokines are likely because, when the tumor enlarges, some cancer cells adapt to the changes that help them escape immunosurveillance [109].

Notably, we also found that, in order to make reasonable predictions regarding the prognosis of cancer patients, it is necessary to study the interactions between immune cells rather than to simply look at the abundance of a certain immune cell type. This observation can be supported by [122], which stated that the immune response following from activation of T cells was dependent on the presence of other immune protagonists, such as macrophages, implying that the interactions between immune cells can affect the immune response.

Our results indicate that cluster 3 had the slowest cancer growth and a relatively low population of cancer cells at the steady state. Meanwhile, cluster 2 had one of the fastest cancer growth rates and, more importantly, the highest number of cancer cells at the steady state. Thus, cluster 3 had the most favorable cancer progression, and cluster 2 had the least favorable cancer progression. These results are in agreement with the findings from clinical data in our previous study in that cluster 3 had the best outcomes and cluster 2 had the worst outcomes [59].

Our global sensitivity analysis shows that the rate at which cytotoxic cells kill cancer cells has a large impact on the growth of osteosarcoma. Therefore, it is probable that treatments that attempt to increase this rate of cytotoxic cells attacking tumor cells, such as PD-1 or CTLA-4 inhibitors, would work well for osteosarcoma. In fact, a phase 2 trial reported that some improvement in cancer progression was observed in osteosarcoma patients treated with the anti PD-1 drug, Pembrolizumab [123]. The combined treatment of PD-1 and CTLA-4 blockade therapy has shown even better responses compared with single checkpoint inhibitors in bone sarcoma [124].

In the mathematical modeling of cancers, one of the main challenges is the large number of unknown parameters and a limited availability of data sets to derive parameters from. To combat this challenge, many mathematical models adopted one or a couple of the following approaches: assuming biologically feasible values for some parameters, using estimated parameters from other diseases or rodent studies, calibrating parameters to fit the biological behaviors from an experimental data set, and varying the parameter values within a reasonable range to study the impact of those parameters on the results.

In our work, we acquired parameter values from experimental studies in the literature and estimated the others using the steady state assumption with the steady state values derived from patient gene expression data. Importantly, we also performed global sensitivity analysis on the estimated parameters.

All mathematical models thus far used the same parameters for all patients, while our model estimates parameters separately for each cluster of patients with distinctive immune compositions. Since patients with different immune compositions have shown different prognoses and different responses to treatment [125,126,127,128,129], estimating the parameters for each cluster separately helps us model the effects of immune cells on cancer growth and their responses to treatment more accurately.

To avoid adding complexity to an already complex network, our study does not model the healthy cells in the tumor microenvironment. While several mathematical models for tumor growth study the competition between healthy cells and cancerous cells [60,130,131,132], these models typically only investigate a small subset of immune cells, unlike our model, which focuses on many important components of the immune system. Moreover, as the cancer self-proliferation rate ($\lambda_{C}$) in our model is taken from osteosarcoma growth data in humans, which is naturally the growth of tumors with the presence of healthy cells, this parameter already encodes the inhibition of cancer growth due to competition with healthy cells. Therefore, even though we do not explicitly model healthy cells, the impact of healthy cells on cancer growth is incorporated implicitly through $\lambda_{C}$.

While it would be ideal to use time course data to derive the parameters in each cluster, the availability of such data is currently limited, and so instead we use the large tumors in each cluster as the steady state values to estimate these parameters. Despite this limitation due to the lack of time course data, our model still provides valuable insights on the progression of osteosarcoma and the impact of the immune system on its growth, and many studies can build upon this one. Ways to improve this model include utilizing partial differential equations to study the growth of osteosarcoma tumors, both in space and in time, or in applying different parameter fitting algorithms [133,134,135,136] to better match the dynamics of the system to real patient data.

Our model provides a foundational work that can be easily adopted by other researchers to determine effective treatment strategies in osteosarcoma. In particular, many cancer treatments, such as chemotherapy, radiotherapy, and hyperthermia, are known to have effects on the immune system. Chemotherapy can be both immunosuppressive and immunostimulatory, depending on the drug and dosage. For example, Cisplatin at high doses can reduce the production of IFN-γ by T cells [137] and suppress the generation of anti-tumor effector cells [138], while Doxorubicin in low doses has been reported to induce immunogenic cell death, leading to the maturation of dendritic cells and the proliferation of cytotoxic T cells [139,140,141,142].

Similar to chemotherapy, radiation can both weaken the immune system by lowering the white blood cell count [143] and enhance anti-tumor immune responses through the release of pro-inflammatory cytokines and tumor antigens [144]. Hyperthermia can activate cytotoxic T cells, dendritic cells, and natural killer cells as well as inhibit immune suppression [145]. Knowing the effects of a treatment on the immune system and cancer cells, one can build upon our model by adding the interactions between the treatment and various components in our network and, thus, find the optimal dosage for a treatment.

## 5. Conclusions

We built a data-driven mathematical model of osteosarcoma progression while taking into account the interactions between immune cells and cancer cells. We determined that, out of the three clusters of osteosarcoma patients with distinct immune compositions, cluster 3 appeared to have the most favorable tumor growth, and cluster 2 had the least favorable growth. During osteosarcoma progression, the number of dendritic cells, helper T cells, cytotoxic cells, and the amount of IFN-γ first increased and then decreased, while the regulatory T cell population decreased and then increased. This switch in the dynamics of immune cells and cytokines happens around the same time that cancer cells have the fastest growth.

The global sensitivity analysis indicated that the cancer death rates by cytotoxic cells and IFN-γ, the cancer proliferation rates by cytokines groups $\mu_{1}$ and $\mu_{2}$, as well as the cancer self-proliferation and apoptosis rates were the most impactful parameters on cancer growth. Additionally, among all immune parameters, the activation and decay rates of macrophages and regulatory T cells had the most impact on cancer growth. This study also shows that it is important to investigate the complex interactions between immune cells and cancer cells instead of purely looking at the abundance of certain immune cells as a marker for the disease’s progression.

## Figures and Tables

**Table 1:** **Model Variables.** Names and descriptions of the variables used in the model.

Variable	Name	Description
$T_{N}$	Naive T-cells	
$T_{h}$	Helper T-cells	
$T_{C}$	Cytotoxic cells	includes CD8+ T-cells and NK cells
$T_{r}$	Regulatory T-cells	
$D_{n}$	Naive dendritic cells	
*D*	Activated dendritic cells	antigen presenting cells
$M_{N}$	Naive macrophages	includes naive macrophages and monocytes
*M*	Macrophages	includes M1 macrophages and M2 macrophages
*C*	Cancer cells	
*N*	Nectrotic cells	
*H*	HMGB1	
$\mu_{1}$	Cytokines group $\mu_{1}$	includes effects of TGF-β, IL-4, IL-10 and IL-13
$\mu_{2}$	Cytokines group $\mu_{2}$	includes effects of IL-6 and IL-17
$I_{\gamma }$	IFN-γ	

**Table 2:** Steady-state abundance of cells and cytokines.

Cluster	$M_{N}^{\infty }$	$M^{\infty }$	$T_{N}^{\infty }$	$T_{h}^{\infty }$	$T_{r}^{\infty }$
1	$6.236\times 10^{6}$	$1.977\times 10^{7}$	$4.926\times 10^{6}$	$7.092\times 10^{6}$	$3.675\times 10^{6}$
2	$3.248\times 10^{7}$	$1.842\times 10^{7}$	$1.047\times 10^{7}$	$1.973\times 10^{6}$	$8.673\times 10^{5}$
3	$1.944\times 10^{7}$	$2.698\times 10^{7}$	$1.368\times 10^{7}$	$1.205\times 10^{6}$	$1.405\times 10^{6}$
	$T_{c}^{\infty }$	$D_{N}^{\infty }$	$D^{\infty }$	$C^{\infty }$	$N^{\infty }$
1	$2.292\times 10^{7}$	$4.826\times 10^{5}$	$9.865\times 10^{5}$	$1.343\times 10^{10}$	$3.764\times 10^{8}$
2	$3.155\times 10^{5}$	$8.927\times 10^{5}$	$7.135\times 10^{5}$	$1.604\times 10^{10}$	$4.257\times 10^{8}$
3	$1.802\times 10^{6}$	$4.591\times 10^{5}$	$3.732\times 10^{5}$	$1.340\times 10^{10}$	$1.544\times 10^{9}$
	$I_{\gamma }^{\infty }$	$\mu_{1}^{\infty }$	$\mu_{2}^{\infty }$	$H^{\infty }$	
1	0.868	21.510	2.067	5.076	
2	0.049	20.714	1.611	4.948	
3	0.263	23.663	1.371	4.453	

**Table 3:** The non-dimensional initial conditions for each cluster.

Cluster	$M_{N}/M_{N}^{\infty }$	$M/M^{\infty }$	$T_{N}/T_{N}^{\infty }$	$T_{h}/T_{h}^{\infty }$	$T_{r}/T_{r}^{\infty }$	$T_{c}/T_{c}^{\infty }$	$D_{N}/D_{N}^{\infty }$
1	2.367	1.005	0.019	0.794	0.764	0.828	1.122
2	0.954	0.753	1.299	1.451	2.313	0.062	0.071
3	0.866	1.104	0.572	0.340	0.484	0	1.643
	$D/D^{\infty }$	$C/C^{\infty }$	$N/N^{\infty }$	$I_{\gamma }/I_{\gamma }^{\infty }$	$\mu_{1}/\mu_{1}^{\infty }$	$\mu_{2}/\mu_{2}^{\infty }$	$H/H^{\infty }$
1	0	0.020	0.160	2.394	1.104	1.806	1.059
2	0.693	0.005	0.018	0.859	1.307	3.259	0.988
3	0	0.014	0.0008	0.276	1.030	1.296	1.284

## Data Availability

Publicly available TARGET data was downloaded from UCSC Xena web portal: https://xenabrowser.net/datapages/, accessed on 16 April 2021. Python scripts for computations and plotting the dynamical results are available here: https://github.com/ShahriyariLab/Data-driven-mathematical-model-of-osteosarcom, accessed on 16 April 2021.

## Abbreviations

The following abbreviations are used in this manuscript:

TARGET	Therapeutically Applicable Research to Generate Effective Treatments
GEO	Gene Expression Omnibus
ODE	Ordinary differential equation
HMGB1	High mobility group box 1
UCSC	University of California Santa Cruz
TIIC	Tumor infiltrating immune cell
NK	Natural killer

## Appendix A. System Analysis

### Appendix A.1. System of ODEs

Combining Equations (1)–(16) we obtain the following system

(A1) $$\begin{array}{cc} \frac{d\left[M_{N}\right]}{dt}=\; & A_{M_{N}}-\left(\lambda_{MI_{\gamma }}\left[I_{\gamma }\right]+\lambda_{M\mu_{1}}\left[\mu_{1}\right]\right)\left[M_{N}\right]-\delta_{M_{N}}\left[M_{N}\right], \end{array}$$

(A2) $$\begin{array}{cc} \frac{d\left[M\right]}{dt}=\; & \left(\lambda_{MI_{\gamma }}\left[I_{\gamma }\right]+\lambda_{M\mu_{1}}\left[\mu_{1}\right]\right)\left[M_{N}\right]-\delta_{M}\left[M\right], \end{array}$$

(A3) $$\begin{array}{cc} \frac{d\left[T_{N}\right]}{dt}=\; & A_{T_{N}}-\left(\lambda_{T_{h}M}\left[M\right]+\lambda_{T_{h}D}\left[D\right]\right)\left[T_{N}\right]\\ \; & -\lambda_{T_{r}\mu_{1}}\left[\mu_{1}\right]\left[T_{N}\right]\\ \; & -\left(\lambda_{T_{c}T_{h}}\left[T_{h}\right]+\lambda_{T_{c}M}\left[M\right]+\lambda_{T_{c}D}\left[D\right]\right)\left[T_{N}\right]-\delta_{T_{N}}\left[T_{N}\right], \end{array}$$

(A4) $$\begin{array}{cc} \frac{d\left[T_{h}\right]}{dt}=\; & \left(\lambda_{T_{h}M}\left[M\right]+\lambda_{T_{h}D}\left[D\right]\right)\left[T_{N}\right]-\left(\delta_{T_{h}T_{r}}\left[T_{r}\right]+\delta_{T_{h}\mu_{1}}\left[\mu_{1}\right]+\delta_{T_{h}}\right)\left[T_{h}\right], \end{array}$$

(A5) $$\begin{array}{cc} \frac{d\left[T_{r}\right]}{dt}=\; & \left(\lambda_{T_{r}\mu_{1}}\left[\mu_{1}\right]\right)\left[T_{N}\right]-\delta_{T_{r}}\left[T_{r}\right], \end{array}$$

(A6) $$\begin{array}{cc} \frac{d\left[T_{c}\right]}{dt}=\; & \left(\lambda_{T_{c}T_{h}}\left[T_{h}\right]+\lambda_{T_{c}M}\left[M\right]+\lambda_{T_{c}D}\left[D\right]\right)\left[T_{N}\right]\\ \; & -\left(\delta_{T_{c}T_{r}}\left[T_{r}\right]+\delta_{T_{c}\mu_{1}}\left[\mu_{1}\right]+\delta_{T_{c}}\right)\left[T_{c}\right], \end{array}$$

(A7) $$\begin{array}{cc} \frac{d\left[D_{N}\right]}{dt}=\; & A_{D_{N}}-\left(\lambda_{DC}\left[C\right]+\lambda_{DH}\left[H\right]\right)\left[D_{N}\right]-\delta_{D_{N}}\left[D_{N}\right], \end{array}$$

(A8) $$\begin{array}{cc} \frac{d\left[D\right]}{dt}=\; & \left(\lambda_{DC}\left[C\right]+\lambda_{DH}\left[H\right]\right)\left[D_{N}\right]-\left(\delta_{DC}\left[C\right]+\delta_{D}\right)\left[D\right], \end{array}$$

(A9) $$\begin{array}{cc} \frac{d\left[C\right]}{dt}=\; & \left(\lambda_{C}+\lambda_{C\mu_{1}}\left[\mu_{1}\right]+\lambda_{C\mu_{2}}\left[\mu_{2}\right]\right)\left[C\right]\left(1-\frac{\left[C\right]}{C_{0}}\right)\\ \; & -\left(\delta_{CT_{c}}\left[T_{c}\right]+\delta_{CI_{\gamma }}\left[I_{\gamma }\right]+\delta_{C}\right)\left[C\right], \end{array}$$

(A10) $$\begin{array}{cc} \frac{d\left[N\right]}{dt}=\; & \alpha_{NC}\left(\delta_{CT_{c}}\left[T_{c}\right]+\delta_{CI_{\gamma }}\left[I_{\gamma }\right]+\delta_{C}\right)\left[C\right]-\delta_{N}\left[N\right], \end{array}$$

(A11) $$\begin{array}{cc} \frac{d\left[I_{\gamma }\right]}{dt}=\; & \lambda_{I_{\gamma }T_{h}}\left[T_{h}\right]+\lambda_{I_{\gamma }T_{c}}\left[T_{c}\right]-\delta_{I_{\gamma }}\left[I_{\gamma }\right], \end{array}$$

(A12) $$\begin{array}{cc} \frac{d\left[\mu_{1}\right]}{dt}=\; & \lambda_{\mu_{1}T_{h}}\left[T_{h}\right]+\lambda_{\mu_{1}M}\left[M\right]+\lambda_{\mu_{1}C}\left[C\right]-\delta_{\mu_{1}}\left[\mu_{1}\right], \end{array}$$

(A13) $$\begin{array}{cc} \frac{d\left[\mu_{2}\right]}{dt}=\; & \lambda_{\mu_{2}T_{h}}\left[T_{h}\right]+\lambda_{\mu_{2}M}\left[M\right]+\lambda_{\mu_{2}C}\left[C\right]-\delta_{\mu_{2}}\left[\mu_{2}\right], \end{array}$$

(A14) $$\begin{array}{cc} \frac{d\left[H\right]}{dt}=\; & \lambda_{HM}\left[M\right]+\lambda_{HD}\left[D\right]+\lambda_{HN}\left[N\right]-\delta_{H}\left[H\right]. \end{array}$$

### Appendix A.2. Positivity

Following [58], to prove that the system with positive coefficients and positive initial conditions has a positive solution, let us consider the set of integrating factors, one for each variable:

$$\begin{array}{cc} \eta_{M_{N}}\left(t\right)=\exp & \int_{0}^{t}\left(\lambda_{MI_{\gamma }}\left[I_{\gamma }\right]+\lambda_{M\mu_{1}}\left[\mu_{1}\right]+\delta_{M_{N}}\right)ds\\ \eta_{T_{N}}\left(t\right)=\exp & \int_{0}^{t}\left(\lambda_{T_{h}M}\left[M\right]+\lambda_{T_{h}D}\left[D\right]+\lambda_{T_{r}\mu_{1}}\left[\mu_{1}\right]\right.\\ \; & \;\left.+\lambda_{T_{c}T_{h}}\left[T_{h}\right]+\lambda_{T_{c}M}\left[M\right]+\lambda_{T_{c}D}\left[D\right]+\delta_{T_{N}}\right)ds \end{array}$$

$$\begin{array}{cc} \eta_{T_{h}}\left(t\right)=\exp & \int_{0}^{t}\left(\delta_{T_{h}T_{r}}\left[T_{r}\right]+\delta_{T_{h}\mu_{1}}\left[\mu_{1}\right]+\delta_{T_{h}}\right)ds\\ \eta_{T_{C}}\left(t\right)=\exp & \int_{0}^{t}\left(\delta_{T_{c}T_{r}}\left[T_{r}\right]+\delta_{T_{c}\mu_{1}}\left[\mu_{1}\right]+\delta_{T_{c}}\right)ds\\ \eta_{D_{N}}\left(t\right)=\exp & \int_{0}^{t}\left(\lambda_{DC}\left[C\right]+\lambda_{DH}\left[H\right]+\delta_{D_{N}}\right)ds\\ \eta_{D}\left(t\right)=\exp & \int_{0}^{t}\left(\delta_{DC}\left[C\right]+\delta_{D}\right)ds\\ \eta_{C}\left(t\right)=\exp & \int_{0}^{t}\left(\delta_{CT_{c}}\left[T_{c}\right]+\delta_{CI_{\gamma }}\left[I_{\gamma }\right]+\delta_{C}-\left(\lambda_{C}+\lambda_{C\mu_{1}}\left[\mu_{1}\right]+\lambda_{C\mu_{2}}\left[\mu_{2}\right]\right)\left(1-\frac{\left[C\right]}{C_{0}}\right)\right)ds\\ \eta_{M}\left(t\right)=\exp & \left(\delta_{M}t\right),\;\eta_{T_{r}}\left(t\right)=\exp\left(\delta_{T_{r}}t\right),\;\eta_{N}\left(t\right)=\exp\left(\delta_{N}t\right),\;\eta_{H}\left(t\right)=\exp\left(\delta_{H}t\right),\\ \eta_{I_{\gamma }}\left(t\right)=\exp & \left(\delta_{I_{\gamma }}t\right),\;\eta_{\mu_{1}}\left(t\right)=\exp\left(\delta_{\mu_{1}}t\right),\;\eta_{\mu_{2}}\left(t\right)=\exp\left(\delta_{\mu_{2}}t\right). \end{array}$$

These integrating factors will not allow us to derive explicit solution as some of them are defined through the unknown variables. However, it is important to note that the factors are strictly positive and allow us to rewrite the system as

$$\begin{array}{cc} \frac{d\left(\left[M_{N}\right]\eta_{M_{N}}\right)}{dt}=\; & A_{M_{N}}\eta_{M_{N}},\\ \frac{d\left(\left[M\right]\eta_{M}\right)}{dt}=\; & \left(\lambda_{MI_{\gamma }}\left[I_{\gamma }\right]+\lambda_{M\mu_{1}}\left[\mu_{1}\right]\right)\left[M_{N}\right]\eta_{M},\\ \frac{d\left(\left[T_{N}\right]\eta_{T_{N}}\right)}{dt}=\; & A_{T_{N}}\eta_{T_{N}},\\ \frac{d\left(\left[T_{h}\right]\eta_{T_{h}}\right)}{dt}=\; & \left(\lambda_{T_{h}M}\left[M\right]+\lambda_{T_{h}D}\left[D\right]\right)\left[T_{N}\right]\eta_{T_{h}},\\ \frac{d\left(\left[T_{r}\right]\eta_{T_{r}}\right)}{dt}=\; & \left(\lambda_{T_{r}\mu_{1}}\left[\mu_{1}\right]\right)\left[T_{N}\right]\eta_{T_{r}},\\ \frac{d\left(\left[T_{c}\right]\eta_{T_{c}}\right)}{dt}=\; & \left(\lambda_{T_{c}T_{h}}\left[T_{h}\right]+\lambda_{T_{c}M}\left[M\right]+\lambda_{T_{c}D}\left[D\right]\right)\left[T_{N}\right]\eta_{T_{c}},\\ \frac{d\left(\left[D_{N}\right]\eta_{D_{N}}\right)}{dt}=\; & A_{D_{N}}\eta_{D_{N}},\\ \frac{d\left(\left[D\right]\eta_{D}\right)}{dt}=\; & \left(\lambda_{DC}\left[C\right]+\lambda_{DH}\left[H\right]\right)\left[D_{N}\right]\eta_{D},\\ \frac{d\left(\left[C\right]\eta_{C}\right)}{dt}=\; & 0,\\ \frac{d\left(\left[N\right]\eta_{N}\right)}{dt}=\; & \alpha_{NC}\left(\delta_{CT_{c}}\left[T_{c}\right]+\delta_{CI_{\gamma }}\left[I_{\gamma }\right]+\delta_{C}\right)\left[C\right]\eta_{N}, \end{array}$$

$$\begin{array}{cc} \frac{d\left(\left[I_{\gamma }\right]\eta_{I_{\gamma }}\right)}{dt}=\; & \left(\lambda_{I_{\gamma }T_{h}}\left[T_{h}\right]+\lambda_{I_{\gamma }T_{c}}\left[T_{c}\right]\right)\eta_{I_{\gamma }},\\ \frac{d\left(\left[\mu_{1}\right]\eta_{\mu_{1}}\right)}{dt}=\; & \left(\lambda_{\mu_{1}T_{h}}\left[T_{h}\right]+\lambda_{\mu_{1}M}\left[M\right]+\lambda_{\mu_{1}C}\left[C\right]\right)\eta_{\mu_{1}},\\ \frac{d\left(\left[\mu_{2}\right]\eta_{\mu_{2}}\right)}{dt}=\; & \left(\lambda_{\mu_{2}T_{h}}\left[T_{h}\right]+\lambda_{\mu_{2}M}\left[M\right]+\lambda_{\mu_{2}C}\left[C\right]\right)\eta_{\mu_{2}},\\ \frac{d\left(\left[H\right]\eta_{H}\right)}{dt}=\; & \left(\lambda_{HM}\left[M\right]+\lambda_{HD}\left[D\right]+\lambda_{HN}\left[N\right]\right)\eta_{H}. \end{array}$$

We see that the right-hand side of each equation in this system is non-negative, which means that the variable-factor product $\left(\left[X\right]\eta_{X}\right)$ is non-decreasing for each variable $\left[X\right]$, and thus, if positive, initially remains positive at all times. Since the integrating factor is positive by design, the positivity of the variables follows.

### Appendix A.3. Boundedness

Similar to [58], we prove the upper bounds on variables after grouping them by types.

#### Appendix A.3.1. Macrophages

Adding Equations (A1) and (A2), we obtain

$$\frac{d\left(\left[M_{N}\right]+\left[M\right]\right)}{dt}=A_{M_{N}}-\delta_{M_{N}}\left[M_{N}\right]-\delta_{M}\left[M\right]\leq A_{M_{N}}-\min\left(\delta_{M_{N}},\;\delta_{M}\right)\left(\left[M_{N}\right]+\left[M\right]\right).$$

Thus, integrating, we obtain

$$\begin{array}{cc} \left[M_{N}\right]+\left[M\right]\leq & \frac{A_{M_{N}}}{\min\left(\delta_{M_{N}},\;\delta_{M}\right)}\left(1-e^{-\min\left(\delta_{M_{N}},\;\delta_{M}\right)t}\right)\\ \; & +e^{-\min\left(\delta_{M_{N}},\;\delta_{M}\right)t}\left(\left[M_{N}\right]\left(0\right)+\left[M\right]\left(0\right)\right). \end{array}$$

Since the right-hand side is bounded and each variable is positive, this proves that each variable is bounded.

#### Appendix A.3.2. T-Cells

Adding Equations (A3)–(A6) and using the positivity of all variables, we obtain

$$\begin{array}{cc} \frac{d\left(\left[T_{N}\right]+\left[T_{h}\right]+\left[T_{r}\right]+\left[T_{c}\right]\right)}{dt}= & A_{T_{N}}-\delta_{T_{N}}\left[T_{N}\right]-\delta_{T_{r}}\left[T_{r}\right]\\ \; & -\left(\delta_{T_{h}T_{r}}\left[T_{r}\right]+\delta_{T_{h}\mu_{1}}\left[\mu_{1}\right]+\delta_{T_{h}}\right)\left[T_{h}\right]\\ \; & -\left(\delta_{T_{c}T_{r}}\left[T_{r}\right]+\delta_{T_{c}\mu_{1}}\left[\mu_{1}\right]+\delta_{T_{c}}\right)\left[T_{c}\right]\\ \leq & A_{T_{N}}-\min\left(\delta_{T_{N}},\;\delta_{T_{h}},\;\delta_{T_{c}},\;\delta_{T_{r}}\right)\left(\left[T_{N}\right]+\left[T_{h}\right]+\left[T_{r}\right]+\left[T_{c}\right]\right). \end{array}$$

Then, integrating, we obtain

$$\begin{array}{cc} \left[T_{N}\right]+\left[T_{h}\right]+\left[T_{r}\right]+\left[T_{c}\right]\leq & \frac{A_{T_{N}}}{\min\left(\delta_{T_{N}},\;\delta_{T_{h}},\;\delta_{T_{c}},\;\delta_{T_{r}}\right)}\left(1-e^{-\min\left(\delta_{T_{N}},\;\delta_{T_{h}},\;\delta_{T_{c}},\;\delta_{T_{r}}\right)t}\right)\\ \; & +e^{-\min\left(\delta_{T_{N}},\;\delta_{T_{h}},\;\delta_{T_{c}},\;\delta_{T_{r}}\right)t}\left(\left[T_{N}\right]\left(0\right)+\left[T_{h}\right]\left(0\right)+\left[T_{r}\right]\left(0\right)+\left[T_{c}\right]\left(0\right)\right). \end{array}$$

Since the right-hand side is bounded and each variable is positive, this proves that each variable is bounded.

#### Appendix A.3.3. Dendritic Cells

Adding Equations (A7) and (A8) and using the positivity of $\left[C\right]$, we obtain

$$\begin{array}{cc} \frac{d\left(\left[D_{N}\right]+\left[D\right]\right)}{dt}= & A_{D_{N}}-\delta_{D_{N}}\left[D_{N}\right]-\left(\delta_{DC}\left[C\right]+\delta_{D}\right)\left[D\right]\\ \leq & A_{D_{N}}-\min\left(\delta_{D_{N}},\;\delta_{D}\right)\left(\left[D_{N}\right]+\left[D\right]\right). \end{array}$$

Similar to the previous cases, integrated bound

$$\left[D_{N}\right]+\left[D\right]\leq \frac{A_{D_{N}}}{\min\left(\delta_{D_{N}},\;\delta_{D}\right)}\left(1-e^{-\min\left(\delta_{D_{N}},\;\delta_{D}\right)t}\right)+e^{\min\left(\delta_{D_{N}},\;\delta_{D}\right)t}\left(\left[D_{N}\right]\left(0\right)+\left[D\right]\left(0\right)\right)$$

proves the upper bound on $\left[D_{N}\right]$ and $\left[D\right]$.

#### Appendix A.3.4. Cancer Cells

Let us rewrite Equation (A9) as follows

$$\begin{array}{cc} \frac{d\left(\left[C\right]-C_{0}\right)}{dt}+ & \frac{\left(\lambda_{C}+\lambda_{C\mu_{1}}\left[\mu_{1}\right]+\lambda_{C\mu_{2}}\left[\mu_{2}\right]\right)\left[C\right]}{C_{0}}\left(\left[C\right]-C_{0}\right)\\ \; & =-\left(\delta_{CT_{c}}\left[T_{c}\right]+\delta_{CI_{\gamma }}\left[I_{\gamma }\right]+\delta_{C}\right)\left[C\right]\leq 0. \end{array}$$

Integrating the inequality with implicit dependence on $\left[C\right],\;\left[\mu_{1}\right],$ and $\left[\mu_{2}\right]$, we obtain

$$\left[C\right]\leq C_{0}-\left(C_{0}-\left[C\right]\left(0\right)\right)\exp\left(-\int_{0}^{t}\frac{\left(\lambda_{C}+\lambda_{C\mu_{1}}\left[\mu_{1}\right]\left(s\right)+\lambda_{C\mu_{2}}\left[\mu_{2}\right]\left(s\right)\right)\left[C\right]\left(s\right)}{C_{0}}ds\right).$$

Since $\left[C\right],\;\left[\mu_{1}\right],$ and $\left[\mu_{2}\right]$ are proven to be positive, the right-hand side is bounded and, thus, $\left[C\right]$ is bounded.

#### Appendix A.3.5. Interferon-γ

We require the bound on interferon before proving the bound on necrotic cells. Since $\left[T_{h}\right]$ and $\left[T_{c}\right]$ are proven to be bounded, we could claim that

$$\lambda_{I_{\gamma }T_{h}}\left[T_{h}\right]+\lambda_{I_{\gamma }T_{c}}\left[T_{c}\right]\leq \lambda_{I_{\gamma }}^{\max}.$$

This, together with Equation (A11), yields the following inequality:

$$\frac{d\left[I_{\gamma }\right]}{dt}+\delta_{I_{\gamma }}\left[I_{\gamma }\right]\leq \lambda_{I_{\gamma }}^{\max},$$

which, when integrated, gives the upper bound on $\left[I_{\gamma }\right]$ as follows:

$$\left[I_{\gamma }\right]\leq \frac{\lambda_{I_{\gamma }}^{\max}}{\delta_{I_{\gamma }}}\left(1-e^{-\delta_{I_{\gamma }}t}\right)+e^{-\delta_{I_{\gamma }}t}\left[I_{\gamma }\right]\left(0\right).$$

#### Appendix A.3.6. Remaining Variables

For each of the remaining variables, the bounds proven above result in the upper bounds for the positive parts of the right-hand side in each equation as follows

$$\begin{array}{cc} \alpha_{NC}\left(\delta_{CT_{c}}\left[T_{c}\right]+\delta_{CI_{\gamma }}\left[I_{\gamma }\right]+\delta_{C}\right)\left[C\right]\leq & \lambda_{N}^{\max},\\ \lambda_{\mu_{1}T_{h}}\left[T_{h}\right]+\lambda_{\mu_{1}M}\left[M\right]+\lambda_{\mu_{1}C}\left[C\right]\leq & \lambda_{\mu_{1}}^{\max},\\ \lambda_{\mu_{2}T_{h}}\left[T_{h}\right]+\lambda_{\mu_{2}M}\left[M\right]+\lambda_{\mu_{2}C}\left[C\right]\leq & \lambda_{\mu_{2}}^{\max},\\ \lambda_{HM}\left[M\right]+\lambda_{HD}\left[D\right]+\lambda_{HN}\left[N\right]\leq & \lambda_{H}^{\max}. \end{array}$$

Then, Equations (A10) and (A12)–(A14) result in the following differential inequalities

$$\begin{array}{cc} \frac{d\left[N\right]}{dt}+\delta_{N}\left[N\right]\leq & \lambda_{N}^{\max},\\ \frac{d\left[\mu_{1}\right]}{dt}+\delta_{\mu_{1}}\left[\mu_{1}\right]\leq & \lambda_{\mu_{1}}^{\max},\\ \frac{d\left[\mu_{2}\right]}{dt}+\delta_{\mu_{2}}\left[\mu_{2}\right]\leq & \lambda_{\mu_{2}}^{\max},\\ \frac{d\left[H\right]}{dt}+\delta_{H}\left[H\right]\leq & \lambda_{H}^{\max}. \end{array}$$

Integrating, we obtain

$$\begin{array}{cc} \left[N\right]\leq & \frac{\lambda_{N}^{\max}}{\delta_{N}}\left(1-e^{-\delta_{N}t}\right)+e^{-\delta_{N}t}\left[N\right]\left(0\right),\\ \left[\mu_{1}\right]\leq & \frac{\lambda_{\mu_{1}}^{\max}}{\delta_{\mu_{1}}}\left(1-e^{-\delta_{\mu_{1}}t}\right)+e^{-\delta_{\mu_{1}}t}\left[\mu_{1}\right]\left(0\right),\\ \left[\mu_{2}\right]\leq & \frac{\lambda_{\mu_{2}}^{\max}}{\delta_{\mu_{2}}}\left(1-e^{-\delta_{\mu_{2}}t}\right)+e^{-\delta_{\mu_{2}}t}\left[\mu_{2}\right]\left(0\right),\\ \left[H\right]\leq & \frac{\lambda_{H}^{\max}}{\delta_{H}}\left(1-e^{-\delta_{H}t}\right)+e^{-\delta_{H}t}\left[H\right]\left(0\right), \end{array}$$

thus, proving the upper bounds.

## Appendix B. Derivation of the Parameter Set

### Appendix B.1. Additional Assumptions

We adopt natural degradation/decay rates of immune cells and cytokines based on information about their half life from the literature (see Table A1). For example, the degradation/decay rate of X is calculated as

$$\begin{array}{c} \delta_{X}=\frac{ln2}{\mathrm{half}\;\mathrm{life}\;\mathrm{of}\;X\;\mathrm{in}\;\mathrm{days}} \end{array}$$

The decay rate of $\mu_{1}$ is estimated to be a weighted average of the decay rates of cytokines within $\mu_{1}$, where the weights are proportional to the abundance of these cytokines. A similar procedure is carried out for $\mu_{2}$. The obtained natural decay rates are as follows:

$$\begin{array}{cccccccc} \delta_{M_{N}}=\; & 0.693,\; & \delta_{M}=\; & 0.015,\; & \delta_{O}=\; & 1.219,\; & \delta_{T_{N}}=\; & 0.00042,\\ \delta_{T_{h}}=\; & 0.231,\; & \delta_{T_{r}}=\; & 0.063,\; & \delta_{T_{c}}=\; & 0.406,\; & \delta_{D_{N}}=\; & 1.664,\\ \delta_{D}=\; & 0.277,\; & \delta_{I_{\gamma }}=\; & 33.27,\; & \delta_{\mu_{1}}=\; & 487.48,\; & \delta_{\mu_{2}}=\; & 5.15,\\ \delta_{H}=\; & 58.7 \end{array}$$

For the proliferation rate of tumor cells, we gathered information on osteosarcoma growth in humans. A study reported that the mean exponential growth constant of primary osteosarcoma tumors was between 0.0054 and 0.02784 [146]. We took the average of these values and chose $\lambda_{C}=0.01662$. Then, we made the assumption that the proliferation rate of cancer cells themselves was 20 times larger than the proliferation rate of cancer for the cytokines group $\mu_{2}$; that is,

(A15) $$\begin{array}{c} \lambda_{C}=\;20\lambda_{C\mu_{2}}\mu_{2}^{\mathrm{mean}} \end{array}$$

$\mu_{2}$ consists of IL-6, which is a major pro-tumor cytokine; therefore, we assume that $\mu_{2}$ is twice as effective at promoting tumor growth compared with $\mu_{1}$:

(A16) $$\begin{array}{cc} \lambda_{C\mu_{2}}\mu_{2}^{\mathrm{mean}}=\; & 2\lambda_{C\mu_{1}}\mu_{1}^{\mathrm{mean}}\\ \mathrm{or}\;\mathrm{equivalently},\;\lambda_{C}=\; & 40\lambda_{C\mu_{1}}\mu_{1}^{\mathrm{mean}} \end{array}$$

We also assume that cytotoxic cells kill tumor cells twice as fast as IFN-γ, and IFN-γ is 10-times more effective at killing cancer cells compared with the cancer cell natural death rate:

(A17) $$\begin{array}{cc} \delta_{CI_{\gamma }}I_{\gamma }^{\mathrm{mean}}=\; & 10\delta_{C} \end{array}$$

(A18) $$\begin{array}{cc} \delta_{CT_{c}}T_{c}^{\mathrm{mean}}=\; & 2\delta_{CI_{\gamma }}I_{\gamma }^{\mathrm{mean}}\;\mathrm{or}\;\delta_{CT_{c}}T_{c}^{\mathrm{mean}}=\;20\delta_{C} \end{array}$$

Since M1 and M2 macrophages are activated solely by IFN-γ and $\mu_{1}$, respectively, we assume that the ratio of macrophages activated by IFN-γ to macrophages activated by $\mu_{1}$ equals to the ratio of M1 to M2 macrophages:

(A19) $$\begin{array}{c} \frac{\lambda_{MI_{\gamma }}I_{\gamma }^{\mathrm{mean}}}{\lambda_{M\mu_{1}}\mu_{1}^{\mathrm{mean}}}=\;\frac{M_{1}^{\mathrm{mean}}}{M_{2}^{\mathrm{mean}}} \end{array}$$

We make the assumption that helper T cells are predominantly activated by antigen presenting dendritic cells and that the inhibition of helper T cells by regulatory T cells and by $\mu_{1}$ are more effective than natural decay:

(A20) $$\begin{array}{cc} \lambda_{T_{h}D}D^{\mathrm{mean}}=\; & 200\lambda_{T_{h}M}M^{\mathrm{mean}} \end{array}$$

(A21) $$\begin{array}{cc} \delta_{T_{h}T_{r}}T_{r}^{\mathrm{mean}}=\; & \delta_{T_{h}\mu_{1}}\mu_{1}^{\mathrm{mean}}=\;20\delta_{T_{h}} \end{array}$$

We also assume that the activation of cytotoxic cells by dendritic cells (both through antigen presentation and IL-12) is twice as effective compared with activation by helper T cells, and four-times as effective compared with activation by macrophages (through IL-12):

(A22) $$\begin{array}{c} \lambda_{T_{c}D}D^{\mathrm{mean}}=\;2\lambda_{T_{c}T_{h}}T_{h}^{\mathrm{mean}}=\;4\lambda_{T_{c}M}M^{\mathrm{mean}} \end{array}$$

while the inhibition of cytotoxic cells by regulatory T cells and by $\mu_{1}$ are each 20-times larger than with natural decay:

(A23) $$\begin{array}{c} \delta_{T_{c}T_{r}}T_{r}^{\mathrm{mean}}=\;\delta_{T_{c}\mu_{1}}\mu_{1}^{\mathrm{mean}}=\;20\delta_{T_{c}} \end{array}$$

For dendritic cells, we make the assumption that activation by HMGB1 is twice as effective compared with activation by cancer cells and that the inhibition by cancer cells is equivalent to the dendritic cells’ innate decay rate:

(A24) $$\begin{array}{cc} \lambda_{DH}H^{\mathrm{mean}}=\; & 2\lambda_{DC}C^{\mathrm{mean}} \end{array}$$

(A25) $$\begin{array}{cc} \delta_{DC}C^{\mathrm{mean}}=\; & \delta_{D} \end{array}$$

Additionally, the following assumptions were used for the production rates of cytokines:

(A26) $$\begin{array}{cc} \lambda_{I_{\gamma }T_{c}}T_{c}^{\mathrm{mean}}=\; & 4\lambda_{I_{\gamma }T_{h}}T_{h}^{\mathrm{mean}} \end{array}$$

(A27) $$\begin{array}{cc} \lambda_{\mu_{1}T_{h}}T_{h}^{\mathrm{mean}}=\; & \lambda_{\mu_{1}M}M^{\mathrm{mean}}=\;\lambda_{\mu_{1}C}C^{\mathrm{mean}} \end{array}$$

(A28) $$\begin{array}{cc} \lambda_{\mu_{2}M}M^{\mathrm{mean}}=\; & \lambda_{\mu_{2}C}C^{\mathrm{mean}}=\;2\lambda_{\mu_{2}T_{h}}T_{h}^{\mathrm{mean}} \end{array}$$

(A29) $$\begin{array}{cc} \lambda_{HN}N^{\mathrm{mean}}=\; & 10\lambda_{HM}M^{\mathrm{mean}}=\;20\lambda_{HD}D^{\mathrm{mean}} \end{array}$$

Lastly, we assume that $\alpha_{NC}=3/4$ and that carrying capacity of cancer is twice the steady state value of cancer, that is $C_{0}=2C^{\infty }$.

### Appendix B.2. Parameter Values and Sources

**Table A1:** Non-dimensional parameter values for each cluster.

Parameter	Cluster 1	Cluster 2	Cluster 3	Source
${\bar{\lambda }}_{MI_{\gamma }}$	$4.3649\times 10^{-3}$	$8.4234\times 10^{-4}$	$2.8083\times 10^{-3}$	Estimated
${\bar{\lambda }}_{M\mu_{1}}$	$1.0635\times 10^{-2}$	$1.4158\times 10^{-2}$	$1.2192\times 10^{-2}$	Estimated
${\bar{\lambda }}_{T_{h}M}$	$3.3434\times 10^{-2}$	$1.9270\times 10^{-2}$	$2.2194\times 10^{-2}$	Estimated
${\bar{\lambda }}_{T_{h}D}$	1.0963×10	7.3778	9.8325	Estimated
${\bar{\lambda }}_{T_{r}\mu_{1}}$	$6.3\times 10^{-2}$	$6.3\times 10^{-2}$	$6.3\times 10^{-2}$	Estimated
${\bar{\lambda }}_{T_{c}T_{h}}$	6.1171	2.3846	2.8415	Estimated
${\bar{\lambda }}_{T_{c}M}$	1.7478	1.2263	1.4683	Estimated
${\bar{\lambda }}_{T_{c}D}$	1.1463×10	9.3900	1.3011×10	Estimated
${\bar{\lambda }}_{DC}$	$4.0114\times 10^{-1}$	$4.8942\times 10^{-1}$	$5.9472\times 10^{-1}$	Estimated
${\bar{\lambda }}_{DH}$	$4.1518\times 10^{-1}$	$4.2621\times 10^{-1}$	$4.1729\times 10^{-1}$	Estimated
$\lambda_{C}$	$1.662\times 10^{-2}$	$1.662\times 10^{-2}$	$1.662\times 10^{-2}$	[146]
${\bar{\lambda }}_{C\mu_{1}}$	$3.7101\times 10^{-4}$	$3.5910\times 10^{-4}$	$4.0692\times 10^{-4}$	Estimated
${\bar{\lambda }}_{C\mu_{2}}$	$7.1405\times 10^{-4}$	$6.3207\times 10^{-4}$	$5.7910\times 10^{-4}$	Estimated
${\bar{\lambda }}_{I_{\gamma }T_{h}}$	6.3095	1.1946×10	4.1848	Estimated
${\bar{\lambda }}_{I_{\gamma }T_{c}}$	2.6961×10	2.1324×10	2.9085×10	Estimated
${\bar{\lambda }}_{\mu_{1}T_{h}}$	$1.8813\times 10^{2}$	$1.1491\times 10^{2}$	$1.0315\times 10^{2}$	Estimated
${\bar{\lambda }}_{\mu_{1}M}$	$1.0751\times 10^{2}$	$1.1818\times 10^{2}$	$1.0661\times 10^{2}$	Estimated
${\bar{\lambda }}_{\mu_{1}C}$	$1.9184\times 10^{2}$	$2.5440\times 10^{2}$	$2.7772\times 10^{2}$	Estimated
${\bar{\lambda }}_{\mu_{2}T_{h}}$	1.2313	$6.8806\times 10^{-1}$	$6.0936\times 10^{-1}$	Estimated
${\bar{\lambda }}_{\mu_{2}M}$	1.4073	1.4153	1.2595	Estimated
${\bar{\lambda }}_{\mu_{2}C}$	2.5113	3.0467	3.2811	Estimated
${\bar{\lambda }}_{HM}$	4.4046	5.8355	1.8254	Estimated
${\bar{\lambda }}_{HD}$	3.6107	5.5856	2.0218	Estimated
${\bar{\lambda }}_{HN}$	5.0685×10	4.7279×10	5.4853×10	Estimated
$\delta_{M_{N}}$	$6.93\times 10^{-1}$	$6.93\times 10^{-1}$	$6.93\times 10^{-1}$	[147,148,149]
$\delta_{M}$	$1.5\times 10^{-2}$	$1.5\times 10^{-2}$	$1.5\times 10^{-2}$	[150,151]
$\delta_{T_{N}}$	$4.2\times 10^{-4}$	$4.2\times 10^{-4}$	$4.2\times 10^{-4}$	[152]
${\bar{\delta }}_{T_{h}T_{r}}$	6.6404	3.1732	5.0991	Estimated
${\bar{\delta }}_{T_{h}\mu_{1}}$	4.1253	3.9929	4.5246	Estimated
$\delta_{T_{h}}$	$2.31\times 10^{-1}$	$2.31\times 10^{-1}$	$2.31\times 10^{-1}$	[153]
$\delta_{T_{r}}$	$6.3\times 10^{-2}$	$6.3\times 10^{-2}$	$6.3\times 10^{-2}$	[154]
${\bar{\delta }}_{T_{c}T_{r}}$	1.1671×10	5.5771	8.9620	Estimated
${\bar{\delta }}_{T_{c}\mu_{1}}$	7.2505	7.0179	7.9524	Estimated
$\delta_{T_{c}}$	$4.06\times 10^{-1}$	$4.06\times 10^{-1}$	$4.06\times 10^{-1}$	[153]
$\delta_{D_{N}}$	1.664	1.664	1.664	[155]
${\bar{\delta }}_{DC}$	$5.3932\times 10^{-1}$	$6.3864\times 10^{-1}$	$7.3501\times 10^{-1}$	Estimated
$\delta_{D}$	$2.77\times 10^{-1}$	$2.77\times 10^{-1}$	$2.77\times 10^{-1}$	[156]
${\bar{\delta }}_{CT_{c}}$	$1.2269\times 10^{-2}$	$9.6574\times 10^{-3}$	$8.4017\times 10^{-3}$	Estimated
${\bar{\delta }}_{CI_{\gamma }}$	$4.5923\times 10^{-3}$	$6.4192\times 10^{-3}$	$8.4660\times 10^{-3}$	Estimated
$\delta_{C}$	$3.0078\times 10^{-4}$	$1.0390\times 10^{-3}$	$2.4530\times 10^{-4}$	Estimated
$\delta_{N}$	$4.5935\times 10^{-1}$	$4.8360\times 10^{-1}$	$1.1137\times 10^{-1}$	Estimated
$\delta_{I_{\gamma }}$	3.327×10	3.327×10	3.327×10	[157]
$\delta_{\mu_{1}}$	$4.8748\times 10^{2}$	$4.8748\times 10^{2}$	$4.8748\times 10^{2}$	[158,159,160,161]
$\delta_{\mu_{2}}$	5.15	5.15	5.15	[162,163]
$\delta_{H}$	5.87×10	5.87×10	5.87×10	[164]
${\bar{A}}_{M_{N}}$	$7.4055\times 10^{-1}$	$7.0151\times 10^{-1}$	$7.1382\times 10^{-1}$	Estimated
${\bar{A}}_{T_{N}}$	$1.0581\times 10^{2}$	1.7917	3.1561	Estimated
${\bar{A}}_{D_{N}}$	3.3325	2.3958	2.4867	Estimated
$\alpha_{M_{N}M}$	3.1701	$5.6721\times 10^{-1}$	1.3878	Scaling factor
$\alpha_{T_{N}T_{h}}$	1.4396	$1.8848\times 10^{-1}$	$8.8053\times 10^{-2}$	Scaling factor
$\alpha_{T_{N}T_{r}}$	$7.4588\times 10^{-1}$	$8.2864\times 10^{-2}$	$1.0267\times 10^{-1}$	Scaling factor
$\alpha_{T_{N}T_{c}}$	4.6531	$3.0144\times 10^{-2}$	$1.3172\times 10^{-1}$	Scaling factor
$\alpha_{D_{N}D}$	2.0440	$7.9922\times 10^{-1}$	$8.1299\times 10^{-1}$	Scaling factor

## Appendix C. Non-Dimensionalization

We obtained the following non-dimensional system:

(A30) $$\begin{array}{cc} \frac{d\left[{\bar{M}}_{N}\right]}{dt}=\; & {\bar{A}}_{M_{N}}-\alpha_{M_{N}M}\left({\bar{\lambda }}_{MI_{\gamma }}\left[{\bar{I}}_{\gamma }\right]+{\bar{\lambda }}_{M\mu_{1}}\left[{\bar{\mu }}_{1}\right]\right)\left[{\bar{M}}_{N}\right]-\delta_{M_{N}}\left[{\bar{M}}_{N}\right] \end{array}$$

(A31) $$\begin{array}{cc} \frac{d\left[\bar{M}\right]}{dt}=\; & \left({\bar{\lambda }}_{MI_{\gamma }}\left[{\bar{I}}_{\gamma }\right]+{\bar{\lambda }}_{M\mu_{1}}\left[{\bar{\mu }}_{1}\right]\right)\left[{\bar{M}}_{N}\right]-\delta_{M}\left[\bar{M}\right] \end{array}$$

(A32) $$\begin{array}{cc} \frac{d\left[{\bar{T}}_{N}\right]}{dt}=\; & {\bar{A}}_{T_{N}}-\alpha_{T_{N}T_{h}}\left({\bar{\lambda }}_{T_{h}M}\left[\bar{M}\right]+{\bar{\lambda }}_{T_{h}D}\left[\bar{D}\right]\right)\left[{\bar{T}}_{N}\right]\\ \; & -\alpha_{T_{N}T_{r}}{\bar{\lambda }}_{T_{r}\mu_{1}}\left[{\bar{\mu }}_{1}\right]\left[{\bar{T}}_{N}\right]\\ \; & -\alpha_{T_{N}T_{c}}\left({\bar{\lambda }}_{T_{c}T_{h}}\left[{\bar{T}}_{h}\right]+{\bar{\lambda }}_{T_{c}M}\left[\bar{M}\right]+{\bar{\lambda }}_{T_{c}D}\left[\bar{D}\right]\right)\left[{\bar{T}}_{N}\right]-\delta_{T_{N}}\left[{\bar{T}}_{N}\right] \end{array}$$

(A33) $$\begin{array}{cc} \frac{d\left[{\bar{T}}_{h}\right]}{dt}=\; & \left({\bar{\lambda }}_{T_{h}M}\left[\bar{M}\right]+{\bar{\lambda }}_{T_{h}D}\left[\bar{D}\right]\right)\left[{\bar{T}}_{N}\right]-\left({\bar{\delta }}_{T_{h}T_{r}}\left[{\bar{T}}_{r}\right]+{\bar{\delta }}_{T_{h}\mu_{1}}\left[{\bar{\mu }}_{1}\right]+\delta_{T_{h}}\right)\left[{\bar{T}}_{h}\right] \end{array}$$

(A34) $$\begin{array}{cc} \frac{d\left[{\bar{T}}_{r}\right]}{dt}=\; & \left({\bar{\lambda }}_{T_{r}\mu_{1}}\left[{\bar{\mu }}_{1}\right]\right)\left[{\bar{T}}_{N}\right]-\delta_{T_{r}}\left[{\bar{T}}_{r}\right] \end{array}$$

(A35) $$\begin{array}{cc} \frac{d\left[{\bar{T}}_{c}\right]}{dt}=\; & \left({\bar{\lambda }}_{T_{c}T_{h}}\left[{\bar{T}}_{h}\right]+{\bar{\lambda }}_{T_{c}M}\left[\bar{M}\right]+{\bar{\lambda }}_{T_{c}D}\left[\bar{D}\right]\right)\left[{\bar{T}}_{N}\right]\\ \; & -\left({\bar{\delta }}_{T_{c}T_{r}}\left[{\bar{T}}_{r}\right]+{\bar{\delta }}_{T_{c}\mu_{1}}\left[{\bar{\mu }}_{1}\right]+\delta_{T_{c}}\right)\left[{\bar{T}}_{c}\right] \end{array}$$

(A36) $$\begin{array}{cc} \frac{d\left[{\bar{D}}_{N}\right]}{dt}=\; & {\bar{A}}_{D_{N}}-\alpha_{D_{N}D}\left({\bar{\lambda }}_{DC}\left[\bar{C}\right]+{\bar{\lambda }}_{DH}\left[\bar{H}\right]\right)\left[{\bar{D}}_{N}\right]-\delta_{D_{N}}\left[{\bar{D}}_{N}\right] \end{array}$$

(A37) $$\begin{array}{cc} \frac{d\left[\bar{D}\right]}{dt}=\; & \left({\bar{\lambda }}_{DC}\left[\bar{C}\right]+{\bar{\lambda }}_{DH}\left[\bar{H}\right]\right)\left[{\bar{D}}_{N}\right]-\left({\bar{\delta }}_{DC}\left[\bar{C}\right]+\delta_{D}\right)\left[\bar{D}\right] \end{array}$$

(A38) $$\begin{array}{cc} \frac{d\left[\bar{C}\right]}{dt}=\; & \left(\lambda_{C}+{\bar{\lambda }}_{C\mu_{1}}\left[{\bar{\mu }}_{1}\right]+{\bar{\lambda }}_{C\mu_{2}}\left[{\bar{\mu }}_{2}\right]\right)\left[\bar{C}\right]\left(1-\frac{\left[\bar{C}\right]}{{\bar{C}}_{0}}\right)\\ \; & -\left({\bar{\delta }}_{CT_{c}}\left[{\bar{T}}_{c}\right]+{\bar{\delta }}_{CI_{\gamma }}\left[{\bar{I}}_{\gamma }\right]+\delta_{C}\right)\left[\bar{C}\right] \end{array}$$

(A39) $$\begin{array}{cc} \frac{d\left[\bar{N}\right]}{dt}=\; & {\bar{\alpha }}_{NC}\left({\bar{\delta }}_{CT_{c}}\left[{\bar{T}}_{c}\right]+{\bar{\delta }}_{CI_{\gamma }}\left[{\bar{I}}_{\gamma }\right]+\delta_{C}\right)\left[\bar{C}\right]-\delta_{N}\left[\bar{N}\right] \end{array}$$

(A40) $$\begin{array}{cc} \frac{d\left[{\bar{I}}_{\gamma }\right]}{dt}=\; & {\bar{\lambda }}_{I_{\gamma }T_{h}}\left[{\bar{T}}_{h}\right]+{\bar{\lambda }}_{I_{\gamma }T_{c}}\left[{\bar{T}}_{c}\right]-\delta_{I_{\gamma }}\left[{\bar{I}}_{\gamma }\right] \end{array}$$

(A41) $$\begin{array}{cc} \frac{d\left[{\bar{\mu }}_{1}\right]}{dt}=\; & {\bar{\lambda }}_{\mu_{1}T_{h}}\left[{\bar{T}}_{h}\right]+{\bar{\lambda }}_{\mu_{1}M}\left[\bar{M}\right]+{\bar{\lambda }}_{\mu_{1}C}\left[\bar{C}\right]-\delta_{\mu_{1}}\left[{\bar{\mu }}_{1}\right] \end{array}$$

(A42) $$\begin{array}{cc} \frac{d\left[{\bar{\mu }}_{2}\right]}{dt}=\; & {\bar{\lambda }}_{\mu_{2}T_{h}}\left[{\bar{T}}_{h}\right]+{\bar{\lambda }}_{\mu_{2}M}\left[\bar{M}\right]+{\bar{\lambda }}_{\mu_{2}C}\left[\bar{C}\right]-\delta_{\mu_{2}}\left[{\bar{\mu }}_{2}\right] \end{array}$$

(A43) $$\begin{array}{cc} \frac{d\left[\bar{H}\right]}{dt}=\; & {\bar{\lambda }}_{HM}\left[\bar{M}\right]+{\bar{\lambda }}_{HD}\left[\bar{D}\right]+{\bar{\lambda }}_{HN}\left[\bar{N}\right]-\delta_{H}\left[\bar{H}\right] \end{array}$$

The non-dimensional parameters are defined as:

$$\begin{array}{cccccc} {\bar{A}}_{M_{N}}=\; & \frac{A_{M_{N}}}{M_{N}^{\infty }},\; & {\bar{A}}_{T_{N}}=\; & \frac{A_{T_{N}}}{T_{N}^{\infty }},\; & {\bar{A}}_{D_{N}}=\; & \frac{A_{D_{N}}}{D_{N}^{\infty }},\\ \alpha_{M_{N}M}=\; & \frac{M^{\infty }}{M_{N}^{\infty }},\; & \alpha_{T_{N}T_{h}}=\; & \frac{T_{h}^{\infty }}{T_{N}^{\infty }},\; & \alpha_{T_{N}T_{r}}=\; & \frac{T_{r}^{\infty }}{T_{N}^{\infty }},\\ \alpha_{T_{N}T_{c}}=\; & \frac{T_{c}^{\infty }}{T_{N}^{\infty }},\; & \alpha_{D_{N}D}=\; & \frac{D^{\infty }}{D_{N}^{\infty }},\; & {\bar{\alpha }}_{NC}=\; & \alpha_{NC}\frac{C^{\infty }}{N^{\infty }},\\ {\bar{C}}_{0}=\; & \frac{C_{0}}{C^{\infty }},\; & {\bar{\lambda }}_{MI_{\gamma }}=\; & \frac{\lambda_{MI_{\gamma }}I_{\gamma }^{\infty }M_{N}^{\infty }}{M^{\infty }},\; & {\bar{\lambda }}_{M\mu_{1}}=\; & \frac{\lambda_{M\mu_{1}}\mu_{1}^{\infty }M_{N}^{\infty }}{M^{\infty }}, \end{array}$$

$$\begin{array}{cccccc} {\bar{\lambda }}_{T_{h}M}=\; & \frac{\lambda_{T_{h}M}M^{\infty }T_{N}^{\infty }}{T_{h}^{\infty }},\; & {\bar{\lambda }}_{T_{h}D}=\; & \frac{\lambda_{T_{h}D}D^{\infty }T_{N}^{\infty }}{T_{h}^{\infty }},\; & {\bar{\lambda }}_{T_{r}\mu_{1}}=\; & \frac{\lambda_{T_{r}\mu_{1}}\mu_{1}^{\infty }T_{N}^{\infty }}{T_{r}^{\infty }},\\ {\bar{\lambda }}_{T_{c}T_{h}}=\; & \frac{\lambda_{T_{c}T_{h}}T_{h}^{\infty }T_{N}^{\infty }}{T_{c}^{\infty }},\; & {\bar{\lambda }}_{T_{c}M}=\; & \frac{\lambda_{T_{c}M}M^{\infty }T_{N}^{\infty }}{T_{c}^{\infty }},\; & {\bar{\lambda }}_{T_{c}D}=\; & \frac{\lambda_{T_{c}D}D^{\infty }T_{N}^{\infty }}{T_{c}^{\infty }},\\ {\bar{\lambda }}_{DC}=\; & \frac{\lambda_{DC}C^{\infty }D_{N}^{\infty }}{D^{\infty }},\; & {\bar{\lambda }}_{DH}=\; & \frac{\lambda_{DH}H^{\infty }D_{N}^{\infty }}{D^{\infty }},\; & {\bar{\lambda }}_{C\mu_{1}}=\; & \lambda_{C\mu_{1}}\mu_{1}^{\infty },\\ {\bar{\lambda }}_{C\mu_{2}}=\; & \lambda_{C\mu_{2}}\mu_{2}^{\infty },\; & {\bar{\lambda }}_{I_{\gamma }T_{h}}=\; & \frac{\lambda_{I_{\gamma }T_{h}}T_{h}^{\infty }}{I_{\gamma }^{\infty }},\; & {\bar{\lambda }}_{I_{\gamma }T_{c}}=\; & \frac{\lambda_{I_{\gamma }T_{c}}T_{c}^{\infty }}{I_{\gamma }^{\infty }},\\ {\bar{\lambda }}_{\mu_{1}T_{h}}=\; & \frac{\lambda_{\mu_{1}T_{h}}T_{h}^{\infty }}{\mu_{1}^{\infty }},\; & {\bar{\lambda }}_{\mu_{1}M}=\; & \frac{\lambda_{\mu_{1}M}M^{\infty }}{\mu_{1}^{\infty }},\; & {\bar{\lambda }}_{\mu_{1}C}=\; & \frac{\lambda_{\mu_{1}C}C^{\infty }}{\mu_{1}^{\infty }},\\ {\bar{\lambda }}_{\mu_{2}T_{h}}=\; & \frac{\lambda_{\mu_{2}T_{h}}T_{h}^{\infty }}{\mu_{2}^{\infty }},\; & {\bar{\lambda }}_{\mu_{2}M}=\; & \frac{\lambda_{\mu_{2}M}M^{\infty }}{\mu_{2}^{\infty }},\; & {\bar{\lambda }}_{\mu_{2}C}=\; & \frac{\lambda_{\mu_{2}C}C^{\infty }}{\mu_{2}^{\infty }},\\ {\bar{\lambda }}_{HM}=\; & \frac{\lambda_{HM}M^{\infty }}{H^{\infty }},\; & {\bar{\lambda }}_{HD}=\; & \frac{\lambda_{HD}D^{\infty }}{H^{\infty }},\; & {\bar{\lambda }}_{HN}=\; & \frac{\lambda_{HN}N^{\infty }}{H^{\infty }},\\ {\bar{\delta }}_{T_{h}T_{r}}=\; & \delta_{T_{h}T_{r}}T_{r}^{\infty },\; & {\bar{\delta }}_{T_{h}\mu_{1}}=\; & \delta_{T_{h}\mu_{1}}\mu_{1}^{\infty },\; & {\bar{\delta }}_{T_{c}T_{r}}=\; & \delta_{T_{c}T_{r}}T_{r}^{\infty },\\ {\bar{\delta }}_{T_{c}\mu_{1}}=\; & \delta_{T_{c}\mu_{1}}\mu_{1}^{\infty },\; & {\bar{\delta }}_{DC}=\; & \delta_{DC}C^{\infty },\; & {\bar{\delta }}_{CT_{c}}=\; & \delta_{CT_{c}}T_{c}^{\infty },\\ {\bar{\delta }}_{CI_{\gamma }}=\; & \delta_{CI_{\gamma }}I_{\gamma }^{\infty }. \end{array}$$

The assumptions (Equations (A15)–(A29)) in non-dimensional form are:

$$\begin{array}{cc} \lambda_{C}=\; & 20{\bar{\lambda }}_{C\mu_{2}}\frac{\mu_{2}^{\mathrm{mean}}}{\mu_{2}^{\infty }}=\;40{\bar{\lambda }}_{C\mu_{1}}\frac{\mu_{1}^{\mathrm{mean}}}{\mu_{1}^{\infty }},\\ {\bar{\delta }}_{CT_{c}}\frac{T_{c}^{\mathrm{mean}}}{T_{c}^{\infty }}=\; & 2{\bar{\delta }}_{CI_{\gamma }}\frac{I_{\gamma }^{\mathrm{mean}}}{I_{\gamma }^{\infty }}=\;20\delta_{C},\\ \frac{{\bar{\lambda }}_{MI_{\gamma }}\frac{I_{\gamma }^{\mathrm{mean}}}{I_{\gamma }^{\infty }}}{{\bar{\lambda }}_{M\mu_{1}}\frac{\mu_{1}^{\mathrm{mean}}}{\mu_{1}^{\infty }}}=\; & \frac{M_{1}^{\mathrm{mean}}}{M_{2}^{\mathrm{mean}}},\\ {\bar{\lambda }}_{T_{h}D}\frac{D^{\mathrm{mean}}}{D^{\infty }}=\; & 200{\bar{\lambda }}_{T_{h}M}\frac{M^{\mathrm{mean}}}{M^{\infty }},\\ {\bar{\delta }}_{T_{h}T_{r}}\frac{T_{r}^{\mathrm{mean}}}{T_{r}^{\infty }}=\; & {\bar{\delta }}_{T_{h}\mu_{1}}\frac{\mu_{1}^{\mathrm{mean}}}{\mu_{1}^{\infty }}=\;20\delta_{T_{h}},\\ {\bar{\lambda }}_{T_{c}D}\frac{D^{\mathrm{mean}}}{D^{\infty }}=\; & 2{\bar{\lambda }}_{T_{c}T_{h}}\frac{T_{h}^{\mathrm{mean}}}{T_{h}^{\infty }}=\;4{\bar{\lambda }}_{T_{c}M}\frac{M^{\mathrm{mean}}}{M^{\infty }},\\ {\bar{\delta }}_{T_{c}T_{r}}\frac{T_{r}^{\mathrm{mean}}}{T_{r}^{\infty }}=\; & {\bar{\delta }}_{T_{c}\mu_{1}}\frac{\mu_{1}^{\mathrm{mean}}}{\mu_{1}^{\infty }}=\;20\delta_{T_{c}},\\ {\bar{\lambda }}_{DH}\frac{H^{\mathrm{mean}}}{H^{\infty }}=\; & 2{\bar{\lambda }}_{DC}\frac{C^{\mathrm{mean}}}{C^{\infty }},\\ {\bar{\delta }}_{DC}\frac{C^{\mathrm{mean}}}{C^{\infty }}=\; & \delta_{D},\\ {\bar{\lambda }}_{I_{\gamma }T_{c}}\frac{T_{c}^{\mathrm{mean}}}{T_{c}^{\infty }}=\; & 4{\bar{\lambda }}_{I_{\gamma }T_{h}}\frac{T_{h}^{\mathrm{mean}}}{T_{h}^{\infty }},\\ {\bar{\lambda }}_{\mu_{1}T_{h}}\frac{T_{h}^{\mathrm{mean}}}{T_{h}^{\infty }}=\; & {\bar{\lambda }}_{\mu_{1}M}\frac{M^{\mathrm{mean}}}{M^{\infty }}=\;{\bar{\lambda }}_{\mu_{1}C}\frac{C^{\mathrm{mean}}}{C^{\infty }},\\ {\bar{\lambda }}_{\mu_{2}M}\frac{M^{\mathrm{mean}}}{M^{\infty }}=\; & {\bar{\lambda }}_{\mu_{2}C}\frac{C^{\mathrm{mean}}}{C^{\infty }}=\;2{\bar{\lambda }}_{\mu_{2}T_{h}}\frac{T_{h}^{\mathrm{mean}}}{T_{h}^{\infty }},\\ {\bar{\lambda }}_{HN}\frac{N^{\mathrm{mean}}}{N^{\infty }}=\; & 10{\bar{\lambda }}_{HM}\frac{M^{\mathrm{mean}}}{M^{\infty }}=\;20{\bar{\lambda }}_{HD}\frac{D^{\mathrm{mean}}}{D^{\infty }}. \end{array}$$
